# l-Isoaspartyl Methyltransferase Deficiency in Zebrafish Leads to Impaired Calcium Signaling in the Brain

**DOI:** 10.3389/fgene.2020.612343

**Published:** 2021-01-21

**Authors:** Remon Soliman, Maria Lorena Cordero-Maldonado, Teresa G. Martins, Mahsa Moein, Jean-François Conrotte, Rebeccah A. Warmack, Alexander Skupin, Alexander D. Crawford, Steven G. Clarke, Carole L. Linster

**Affiliations:** ^1^Luxembourg Centre for Systems Biomedicine, University of Luxembourg, Esch-sur-Alzette, Luxembourg; ^2^Department of Chemistry and Biochemistry, The Molecular Biology Institute, University of California, Los Angeles, Los Angeles, CA, United States; ^3^University of California, San Diego, La Jolla, CA, United States; ^4^Department of Preclinical Sciences and Pathology, Norwegian University of Life Sciences, Oslo, Norway; ^5^Institute for Orphan Drug Discovery, Bremer Innovations- und Technologiezentrum, Bremen, Germany

**Keywords:** isoaspartyl, protein repair, zebrafish, calcium signaling, HT22 cells

## Abstract

Isomerization of l-aspartyl and l-asparaginyl residues to l-isoaspartyl residues is one type of protein damage that can occur under physiological conditions and leads to conformational changes, loss of function, and enhanced protein degradation. Protein l-isoaspartyl methyltransferase (PCMT) is a repair enzyme whose action initiates the reconversion of abnormal l-isoaspartyl residues to normal l-aspartyl residues in proteins. Many lines of evidence support a crucial role for PCMT in the brain, but the mechanisms involved remain poorly understood. Here, we investigated PCMT activity and function in zebrafish, a vertebrate model that is particularly well-suited to analyze brain function using a variety of techniques. We characterized the expression products of the zebrafish *PCMT* homologous genes *pcmt* and *pcmtl*. Both zebrafish proteins showed a robust l-isoaspartyl methyltransferase activity and highest mRNA transcript levels were found in brain and testes. Zebrafish morphant larvae with a knockdown in both the *pcmt* and *pcmtl* genes showed pronounced morphological abnormalities, decreased survival, and increased isoaspartyl levels. Interestingly, we identified a profound perturbation of brain calcium homeostasis in these morphants. An abnormal calcium response upon ATP stimulation was also observed in mouse hippocampal HT22 cells knocked out for *Pcmt1*. This work shows that zebrafish is a promising model to unravel further facets of PCMT function and demonstrates, for the first time *in vivo*, that PCMT plays a pivotal role in the regulation of calcium fluxes.

## Introduction

Protein l-isoaspartyl methyltransferase (PCMT) is an enzyme that specifically recognizes isoaspartyl residues in proteins, methylates them and initiates their reconversion to normal aspartyl residues (Johnson et al., 1987b). Isoaspartyl residues are formed through a nucleophilic attack of the nitrogen atom in the C-flanking peptide bond on the side chain carbonyl carbon atom of an aspartyl or asparaginyl residue. This leads to the formation of an unstable succinimide intermediate, which hydrolyses to a mixture of aspartyl and isoaspartyl residues, with the latter being the major product (70%) (Geiger and Clarke, 1987). PCMT catalyzes the transfer of a methyl group from the cofactor *S*-adenosyl methionine (SAM) to the free carboxyl group of isoaspartyl residues, accelerating the formation of the succinimide intermediate and thereby favoring repair toward the normal aspartyl residue (Johnson et al., 1987b; Skinner et al., 2000). Previous findings indicate that isoaspartyl formation and the PCMT repair mechanism are involved in neurodegenerative disorders, including Alzheimer's disease (Watanabe et al., 1999; Shimizu et al., 2000; Yang et al., 2011; Warmack et al., 2019), Parkinson's disease (Vigneswara et al., 2006, 2013; Morrison et al., 2012) and multiple sclerosis (Friedrich et al., 2016). For instance, some of the hallmark proteins aggregating in these diseases (beta-amyloid, tau, alpha-synuclein, and myelin basic protein) were found to be prone to isoaspartyl damage accumulation. Therefore, a deeper understanding of the implications of PCMT-mediated protein repair may suggest new routes for disease modulation, especially in these neurological disorders.

PCMT is widely conserved across species except for a few Gram-positive bacteria and fungal species, including *Saccharomyces cerevisiae* (Patananan et al., 2014). Various PCMT-deficient cellular and animal models (including *Escherichia coli, Caenorhabditis elega*ns*, Drosophila melanogaster*, and mice) have been generated to elucidate facets of the physiological function of PCMT, notably its crucial role for normal brain function and its interplay with various signaling pathways (Kagan et al., 1997b; Kim E. et al., 1997; Chavous et al., 2001; Kindrachuk et al., 2003; Khare et al., 2011). *Pcmt1* knockout mice die pre-maturely (on average 2 months after birth) and experience massive seizures during their short life (Kim E. et al., 1997). Tissue specific rescue of PCMT expression under the control of a neuronal promoter in these knockout mice led to reduced seizures and extended lifespan (Kim E. et al., 1997; Lowenson et al., 2001), further supporting the crucial role of PCMT specifically in the brain. In addition, PCMT, while ubiquitously expressed in all tissues, is widely reported to exhibit highest expression levels in the brain (Boivin et al., 1995; Kim E. et al., 1997; Mizobuchi et al., 2008). Although the catalytic function of PCMT is known, its physiological roles in the brain remain less well-understood and new animal models may allow further progress in that direction.

The *Pcmt1* knockout mice allowed the identification of many physiological substrates of this protein repair enzyme. Among those, one exceptionally good substrate, calmodulin, was also one of the first proteins whose function was proven to be affected by isoaspartyl formation (Gagnon et al., 1981; Johnson et al., 1987a; Ota and Clarke, 1990). In addition, looking for PCMT binding partners using a yeast two-hybrid screen, calmodulin was found to interact with PCMT not only as a substrate, but also as an activator of its methyltransferase activity (O'Connor and O'Connor, 1998). Despite these observations and the identification of several other calcium-binding proteins as PCMT substrates [e.g., calreticulin and S100A4 (Vigneswara et al., 2006; Xia et al., 2017)], the direct effect of PCMT on calcium signaling in cells or organisms remains elusive.

Calcium signaling is an important component of brain function, for instance during neuronal differentiation, release of neurotransmitters and apoptosis (Kostyuk and Verkhratsky, 1994; Verkhratsky and Kettenmann, 1996). The currently available vertebrate PCMT knockout model (*Pcmt1* knockout mice) does not allow easy access to the brain for calcium imaging. By contrast, the transparency of zebrafish (*Danio rerio*) at early developmental stages allows for *in vivo* imaging of the brain, suggesting that this organism may serve as an excellent alternative vertebrate model for further functional analyses of PCMT. For calcium fluxes in particular, a powerful approach consists in the use of zebrafish larvae expressing a genetically encoded calcium indicator in which brain calcium transients can be monitored *in vivo*. Zebrafish has been used before as a model organism to investigate neurological disorders, including epilepsy and neurodegenerative diseases (Baraban, 2007; Kabashi et al., 2011; Martín-Jiménez et al., 2015; Soman et al., 2017; Liu and Baraban, 2019). In this study, we aimed at developing a zebrafish model deficient in PCMT to further unravel the physiological function of this protein, notably with regards to calcium signaling. As previously suggested by *in vitro* evidence, our results in zebrafish, but also in a *Pcmt1* knockout mouse hippocampal cell line, provide additional support for an important role of isoaspartyl repair in brain calcium homeostasis in the living cell and a whole organism model.

## Materials and Methods

### Reagents

Reagents, of analytical grade whenever possible, were acquired from Sigma-Aldrich (St. Louis, MO, USA), unless otherwise indicated. Primers for cloning and qPCR, desalted grade, were ordered from Eurogentec (Liège, Belgium) (Supplementary Table 1).

### Zebrafish Strains and Husbandry

Adult zebrafish and embryos were raised and maintained in the LCSB Aquatic Facility at 28 (±0.5)°C with light/dark cycles of 14/10 h, respectively, according to standard protocols (Westerfield, 2000). Embryos were obtained by natural spawning and after collection and elimination of unfertilized eggs, they were reared in 0.3 × Danieau's solution (17 mM NaCl, 2 mM KCl, 0.12 mM MgSO_4_, 1.8 mM Ca(NO_3_)_2_, 1.5 mM HEPES pH 7.5 and 1.2 μM methylene blue) until needed for experiments.

The zebrafish strains used were: AB (wild-type), *nacre* (*mitfa* mutant), and *beta-actin*:GCaMP6f (transgenic line). The latter, kindly provided by Dr. Francesca Peri (EMBL, Heidelberg, Germany), ubiquitously expresses calmodulin (CaM) fused to a circularly permuted green fluorescent protein (GFP) through an M13 peptide in the cytoplasm, under the control of the *beta-actin* promoter (Muto and Kawakami, 2011). Upon binding of calcium to CaM, the CaM/M13 domain undergoes conformational changes leading to increased fluorescence of GFP. As transparency is essential to obtain a good visualization of fluorescent signal, the *beta-actin*:GCaMP6f strain was crossed with the *nacre* strain and their non-pigmented progeny was selected for the experiments.

### Ethics Statement

The Aquatic Facility at the Luxembourg Centre for Systems Biomedicine is registered as an authorized breeder, supplier and user of zebrafish with Grand-Ducal decree of 20 January 2016. All practices involving zebrafish were performed in accordance with European laws, guidelines and policies for animal experimentation, housing and care (European Directive 2010/63/EU on the protection of animals used for scientific purposes). The present study did not involve any procedure within the meaning of Article 3 of the Directive 2010/63/EU and as such it was not subjected to authorization by an ethics committee.

### Gene Knock Down by Morpholino Injection in Zebrafish

Splice blocking morpholino oligomers (MOs) targeting zebrafish *pcmt* and *pcmtl* were designed by GeneTools LLC (Philomath, OR, USA) and had the following sequences: *pcmt*-i2e3, 5′-GCTTGATAACCTGCATGAAATGAAC-3′; *pcmtl*-e1i1, 5′-AGAGCGGAGCAAAAATCTTACTCGT-3′; *pcmt*-e5i5, 5′-AAGATGGATGAGCAGACCAACCGAT-3′; *pcmtl*-e4i4, 5′-CATGTGTTTTTCTGACCTACCATAT-3′. A MO targeting human beta-globin (5′-CCTCTTACCTCAGTTACAATTTATA-3′) served as a negative control. MO stock solutions (1 mM) containing 0.1% phenol red were loaded into glass capillaries pulled in house. Pressure and time of injection were adjusted to inject 0.15 mm diameter droplets (about 2 nl) into eggs at 1- to 2-cell stage. The injected eggs were maintained at 28.5°C with standard light/dark cycle and with daily medium exchange until used for experiments.

### Exogenous Expression of *pcmt* and *pcmtl* in Zebrafish

To restore *pcmt* and *pcmtl* expression in morphant zebrafish larvae, we either injected the corresponding mRNAs or plasmids containing the coding sequences together with transposase mRNA for genome integration (Kwan et al., 2007). For the first approach, both *pcmt* and *pcmtl* were cloned separately into pCS2+ plasmids downstream of the SP6 promoter. Linearized plasmids were used for *in vitro* transcription (IVT) using the mMESSAGE mMACHINE™ SP6 Transcription Kit (Thermo Fisher Scientific, Waltham, MA, USA) and following the supplier's instructions. Residual plasmid DNA was removed by the addition of 1 μl TURBO DNase (supplied in the aforementioned kit) and incubation at 37°C for 15 min. Finally, transcribed RNA was purified using the MEGAclear™ kit (Ambion, Austin, TX, USA). Rescue experiments were performed by co-injection of 50 pg of both *pcmt* and *pcmtl* transcripts together with *pcmt* e5i5 and *pcmtl* e4i4 MOs. For the second approach, the *pcmt* and *pcmtl* coding sequences were cloned separately into pGEM-T plasmids downstream of an *ef1*α promoter and trapped between two *Tol2* sites (Kwan et al., 2007). Transposase mRNA was produced by IVT as described above. For rescue experiments, 100 pg of each recombinant *Tol2* plasmid was co-injected with 50 pg transposase mRNA into one-cell stage zebrafish embryos.

### Mammalian Cell Culture

The mouse hippocampal cell line HT22 (Liu et al., 2009) was maintained in Dulbecco's Modified Eagle Medium (DMEM, Thermo Fisher Scientific, Waltham, MA, USA), supplemented with 10% (v/v) fetal bovine serum (FBS) and 1% (v/v) Penicillin-Streptomycin (final concentrations of 100 units/ml and 100 μg/ml for penicillin and streptomycin, respectively). Cells were stored as 1-ml aliquots (10^6^ cells) in medium supplemented with 5% DMSO at −150°C. For experiments, cells were thawed and washed with culture medium to remove DMSO. Cells were then cultivated at 37°C and 5% CO_2_ and passaged 3 times a week. Experiments were performed with cells that had been passaged 10–28 times.

### *Pcmt1* Knockout in Mouse HT22 Cells Using CRISPR/Cas9

The *Pcmt1* gene was knocked out in HT22 cells, using a CRISPR/Cas9-based method described by Ran et al. (2013). Two gRNAs spanning 40 bp in the mouse *Pcmt1* gene were designed using the CRISPR finder tool from the Sanger Institute^1^. The gRNA sequences were 5′GCCGCACTCGCCCCGAGGAC3′ (gRNA 1) and 5′GTGAGTGGCAGAACGCTTAC3′ (gRNA 2). A scrambled sequence of gRNA 1 was used as a control. The three gRNAs were cloned into the pX459 plasmid (Addgene plasmid #48139). HT22 cells were transfected with recombinant plasmid using Lipofectamine 2000 (Thermo Fisher Scientific, Waltham, MA, USA) following the standard transfection protocol provided by the manufacturer. Puromycin was added 1 day after transfection at a final concentration of 1 μg/ml for selection of plasmid containing cells. Medium was refreshed after 3 days and 3 days later puromycin-resistant cells were trypsinized, counted and diluted to a density of 1 cell per 200 μl medium. Single resistant cells were seeded in each well of a 96-well plate followed by clone expansion for 1 week. Several wells with single clones were selected for genotyping. A primer pair (Supplementary Table 1) spanning the two target sites was used to validate the 40 bp-deletion in the *Pcmt1* gene. Control cells were transfected with the plasmid carrying the scrambled gRNA and processed in parallel to the Pcmt1 knockout cells. Clones from the control cells (wild-type *Pcmt1* amplicon) and knockout cells (40 bp deletion in *Pcmt1*) were propagated and stored at −150°C in DMEM supplemented with 5% DMSO.

### Recombinant Protein Expression and Purification

The open reading frames (ORFs) of zebrafish *pcmt* and *pcmtl* were PCR-amplified from clones IRBOp991B0677D and IRBOp991C012D (SourceBioscience, UK), respectively. Both ORFs were cloned separately into the pET100 plasmid (Thermo Fisher Scientific, Waltham, MA, USA), leading to the addition of a 36 amino acid N-terminal sequence (MRGSHHHHHH GMASMTGGQQ MGRDLYDDDD KDHPFT), including the polyhistidine region, the Xpress™ epitope and the Enterokinase cleavage site. Recombinant proteins were expressed in *E. coli* BL21(DE3) cells (Thermo Fisher Scientific, Waltham, MA, USA) at 18°C with continuous shaking for 10 h, after induction by addition of 0.1 mM isopropyl-1-thio-β-d-galactopyranoside (IPTG) to bacterial cultures having reached an OD_600_ of 0.7. Cells were harvested by a 20-min centrifugation at 4816 × *g* and 4°C and pellets were stored at −80°C until further use.

For protein extraction, pellets were resuspended in 50 ml lysis buffer [25 mM Tris-HCl pH 8.0, 300 mM NaCl, 0.5 mM PMSF, 1 mM DTT, 0.1 mg/ml lysozyme, 1 × protease inhibitor cocktail plus EDTA (Roche, Basel, Switzerland)] and sonicated using a BRANSON Digital Sonifier 200W S-250D (3 cycles separated by 1-min breaks; each cycle lasted 20 s, alternating 0.5 s “on” and 1 s “off” periods at 50% amplitude). Lysates were centrifuged for 40 min at 15 000 × *g* and 4°C and the supernatant was filtered through a Minisart 5 μm cellulose acetate membrane (Sartorious, France).

An Äkta purifier system (GE Healthcare, Chicago, IL, USA) was used for protein purification. The filtered protein extract (about 50 ml) was loaded onto a 1-ml HisTrap column (GE Healthcare, Chicago, IL, USA), previously equilibrated with buffer A (25 mM Tris-HCl pH 8, 300 mM NaCl, 10 mM imidazole), at a constant flow rate of 1 ml/min. After washing the column with buffer A, a second washing step followed with buffer A supplemented with 25 mM imidazole. Finally, His-tagged proteins were eluted with a linear gradient of imidazole (25–300 mM) applied over 20 min with collection of 1 ml fractions. The fractions showing high protein concentration and high purity of the protein of interest by SDS-PAGE analysis were pooled, dialyzed overnight against a buffer containing 20 mM Tris-HCl pH 7.6, 25 mM NaCl and 20% glycerol (2 × 2L), and stored at −20°C until further analysis.

### Real Time qPCR

RNA extraction was performed from either zebrafish larvae, organs, or mammalian cells using TriPure reagent (Roche, Basel, Switzerland) following the manufacturer's protocol. Briefly, 1 ml TriPure reagent was added to 15 larvae, dissected organs from 2-year old adult zebrafish or to wells containing 70% confluent cells in 6-well plates, followed by sample homogenization. Zebrafish larvae or organs were homogenized using a cooled Precellys24 (Bertin Instruments, Montigny-le-Bretonneux, France); 3 cycles of 10 s at 6,000 rpm separated by 15 s breaks) whereas mammalian cells were homogenized by pipetting up and down. After centrifugation, RNA was precipitated from the supernatants by addition of isopropanol, the pellets washed with 80% ethanol and dissolved in nuclease-free water. 1 μg RNA was used for cDNA synthesis using the RevertAid H Minus First Strand cDNA synthesis kit (Thermo Fisher Scientific, Waltham, MA, USA). The cDNA was diluted 4-fold and 2 μl were used for qPCR analysis in a total volume of 20 μl using iQ SYBR green mix (Biorad Laboratories, Hercules, CA, USA). The qPCR reactions were performed in a LightCycler 480 (Roche, Basel, Switzerland) with the following parameters: 5 min at 95°C, followed by 40 cycles of 30 s at 95°C, 30 s at 60°C, and 30 s at 72°C, and finally acquisition of a melting curve. Relative fold changes were calculated based on three biological replicates using the 2^−Δ*ΔCt*^ method and *eef1a1l1* (Tang et al., 2007) as the reference gene.

### Protein Extraction From Zebrafish Larvae and HT22 Cells

Before protein extraction from zebrafish larvae, deyolking was done according to a method described by Link et al. (2006). About 100 larvae at 2 or 4 days post-fertilization (dpf) were collected in a tube containing 200 μl of deyolking buffer (55 mM NaCl, 1.8 mM KCl, 1.25 mM NaHCO_3_). Larvae were pipetted up and down with a tip cut off at the extremity to match approximately the diameter of the yolk and favor yolk disruption through shear stress. Samples were then washed 3 times with 200 μl wash buffer (110 mM NaCl, 3.5 mM KCl, 2.7 mM CaCl_2_, 10 mM Tris-HCl pH 8.5) and centrifuged briefly to collect the larvae. Pellets were stored at −80°C until used. For protein extraction, 200 μl of lysis buffer (50 mM Tris-HCl pH 7.5, 150 mM NaCl, 1% Triton X-100, 0.1% SDS, 1 mM EDTA, 1 × protease inhibitor cocktail) were added to the deyolked larvae. Tissue disruption was carried out using a cooled Precellys-24 homogenizer (3 cycles of vigorous shaking at 3,000 rpm for 10 s followed by a 15-s break) (Bertin Instruments, Montigny-le-Bretonneux, France). Samples were then incubated on ice for 30 min and centrifuged at 16,100 × *g* for 20 min at 4°C.

For the HT22 cell line, cells were seeded in 6-well plates and incubated for 2 days under standard cultivation conditions. For protein extraction, cells were scraped into 0.2 M Bis-Tris buffer pH 6 supplemented with protease inhibitor cocktail, and lysed by three freeze-thaw cycles. The lysates were centrifuged and the supernatants used for measurements of isoaspartyl residues (or the isoaspartyl methyltransferase activity) by the methanol diffusion assay.

Protein concentrations in zebrafish and cell extracts were determined using the Bradford assay.

### SDS-PAGE and Western Blotting

Protein extracts or purified protein samples were mixed with SDS sample buffer (63 mM Tris-HCl pH 6.8, 2% w/v SDS, 10% v/v glycerol, 0.002% w/v bromophenol blue, and 5% β-mercaptoethanol) and heated at 95°C for 5 min. Unless otherwise mentioned, the equivalent of 20 μg protein of each sample was loaded into pre-cast SDS-PAGE gels (BioRad Laboratories, Hercules, CA, USA). Separation was achieved at a constant voltage (160 V) for 1 h using migration buffer (25 mM Tris pH 8.3, 192 mM glycine and 1% SDS). The gels were either used for protein detection by Coomassie blue staining or for Western blot analysis.

For Western blotting, proteins were transferred to a PVDF membrane using a semi-dry method. This membrane was washed briefly with TBS-T buffer (20 mM Tris-HCl pH 6.7, 137 mM NaCl, 0.1% Tween20) and blocked with 5% BSA solution (1 h at room temperature on an orbital shaker). The membrane was then incubated overnight at 4°C and shaking with the primary antibody (rabbit anti-PCMT1, #ab97446, Abcam, Cambridge, UK) diluted 1:1,000 in TBS-T buffer containing 1% BSA, followed by three washing steps with TBS-T buffer for 5 min. Finally, the secondary antibody (goat anti-rabbit IgG coupled to HRP, #7074, Cell Signaling Technology, Danvers, MA, USA), diluted 1:5,000 in TBS-T containing 1% BSA, was incubated with the membrane for 1 h at room temperature, followed by washes with TBS-T buffer. Protein bands were detected using ECL reagents (#RPN2232, Amersham, UK) and an Odyssey FC Imaging System (Li-COR). Band intensity was quantified using the Image Studio™ software (Li-COR, Lincoln, NE, USA) with exposure times ranging from 0.5 to 2 min.

### Isoaspartyl Methyltransferase Activity Assay

For kinetic characterization of the purified *D. rerio* Pcmt and Pcmtl enzymes, a methanol vapor diffusion assay was applied as adapted from Patananan et al. (2014). Reaction mixtures, incubated at 30°C, contained 0.2 M Bis-Tris pH 6.0, 2 μg of recombinant Pcmt or Pcmtl, 14.5 μM *S*-adenosyl methionine (SAM) and different concentrations (0.2–60 μM) of the isoaspartyl substrate KASAisoDLAKY (synthesized by Bachem, Switzerland) in a total volume of 50 μl. The SAM cofactor solution was prepared by diluting 1 part of 7 μM [methyl-^3^H]SAM [PerkinElmer, UK; 78 Ci/mmol, 0.55 μCi/μl in 10 mM H_2_SO_4_:ethanol (9:1, v/v)] in 9 parts 80 μM non-labeled SAM (Sigma-Aldrich, St. Louis, MO, USA). Reactions were stopped by addition of 10 μl 0.2 M NaOH at different time points to determine initial velocities. 100 μl of the stopped reaction mixture were spotted on a filter paper (1 × 2 cm) and placed above 2 ml liquid scintillation cocktail (Ultima Gold XR, Perkin Elmer) in a 4-ml vial. The closed vial was incubated for 4 h at room temperature to allow the diffusion of radiolabelled methanol into the liquid scintillation cocktail. Finally, the filter paper was removed and vials were placed into a MicroBeta2 plate counter (PerkinElmer, UK). Counts per minute (cpm) were converted to moles of methylated isoaspartyl residues based on a standard curve prepared by using the same method with an excess of recombinant human protein l-isoaspartyl methyltransferase (rhPCMT1; 5 μg) and known amounts of the KASAisoDLAKY peptide. Kinetic parameters were estimated using non-linear regression fitting with GraphPad Prism (version 7.04).

In zebrafish larvae and HT22 cell extracts, the isoaspartyl methyltransferase activity was assayed as described above, except that the reaction mixture contained 100 μM of KASAisoDLAKY peptide, 10–30 μg protein extract as the isoaspartyl methyltransferase source, and was incubated at 30°C for 2 h before stopping the reaction by addition of NaOH. Zebrafish and HT22 protein extracts were prepared as described above.

### Isoaspartyl Level Determination in Protein Extracts

The methanol vapor diffusion assay was also applied to measure the amount of isoaspartyl residues in protein extracts prepared from mammalian cells and zebrafish larvae. The assay mixture was prepared and treated as described above for the PCMT activity assay, with 10–30 μg total protein and 5 μg rhPCMT1, and a 2-h incubation at 30°C before terminating the reaction by alkalinisation and spotting on filter papers.

We also analyzed the l-isoaspartyl content of zebrafish protein extracts by SDS-PAGE fluorography, as described previously (Patananan et al., 2014). Briefly, 15 μg of total zebrafish proteins were incubated for 2 h at 37°C in a 30 μL reaction mixture containing 0.2 M Bis-Tris-HCl, pH 6.0, 4 μg rhPCMT, and 0.3 μM S-adenosyl-L-[methyl-^3^H] methionine (PerkinElmer, UK; 75–85 Ci/mmol). The reaction was stopped by adding 5 μL SDS-PAGE loading buffer (250 mM Tris-HCl, pH 6.8, 10% (w/v) SDS, 50% (v/v) glycerol, 5% (v/v) β-mercaptoethanol, and 0.05% (w/v) bromophenol blue). Samples were heated at 100°C for 3 min and separated on a 4–12% polyacrylamide ExpressPlus PAGE gel (Genscript Biotech, Piscataway, NJ, USA), followed by staining with Coomassie Blue. For fluorography, gels were subsequently incubated with EN^3^HANCE solution (PerkinElmer, UK) for 1 h, rinsed in water for 30 min, and dried before film exposure (8 × 10-inch Hyblot Cl, Denville Scientific, Metuchen, NJ, USA) for 7 days at −80°C.

### Calcium Imaging in Zebrafish

Eggs collected from *beta-actin*:GCaMP6f transgenic zebrafish were injected at 1–2 cell stage with either control MO, *pcmt*-e5i5 and *pcmtl*-e4i4 MOs (*pcmt*/*l* MOs) or *pcmt*/*l* MOs together with rescue plasmids. Embryos were incubated at 28.5°C until 4 dpf when calcium imaging was performed. Prior to imaging, larvae were incubated in 20 mM pentylenetetrazol (PTZ) for 30 min. Larvae were then immobilized in 1.3% low melting agarose prepared in 0.3 × Danieau's without methylene blue on a glass bottom dish (MatTek, MA, USA) and covered with Danieau's supplemented with 20 mM PTZ to avoid dryness of the gel. The immobilized larvae were imaged for 30 min using a Nikon SMZ25 stereomicroscope using a Nikon SHR Plan Apo 2 × WD:60 lens under illumination by an epi-fluorescence light source with a GFP-B filter cube (Nikon). Images were acquired at 2 Hz with 500 ms exposure time, 640 × 512 resolution, 8 Bit depth, and 5 × zoom. The mean fluorescence intensity (F) of the brain over time was analyzed using the Fiji software and normalized using the CaSiAn software (Moein et al., 2018) for ΔF/F_0_ analysis.

### Calcium Imaging in HT22 Cells

*Pcmt1* knockout or control cells were seeded in 12-well plates at a density of 25,000 cells per well and incubated for 2 days. Cells were then washed with PBS and 1 ml of fresh medium was added (DMEM without phenol red and supplemented with 25 mM glucose, 4 mM glutamine, 10% FBS and 1% Penicillin-Streptomycin). Cells were stained by adding 250 μl of Fluo-4 AM (Gee et al., 2000) (# F10471, Thermo Fisher Scientific, Waltham, MA, USA) reagent into each well followed by 30 min incubation at 37°C and 5% CO_2_. Cells were then stimulated by addition of ATP at final concentrations of 20–40 μM and calcium signals were acquired using a Nikon Ti Eclipse inverted microscope in a controlled environment (37°C and 5% CO_2_) with excitation at 490 to 510 nm and sample rate of 0.33 Hz. Signals were extracted from acquired images using ImageJ and analyzed using the CaSiAn software (Moein et al., 2018).

## Results

### Zebrafish Expresses Two PCMT Paralogs From the Earliest Developmental Stages

**Figure 1:**
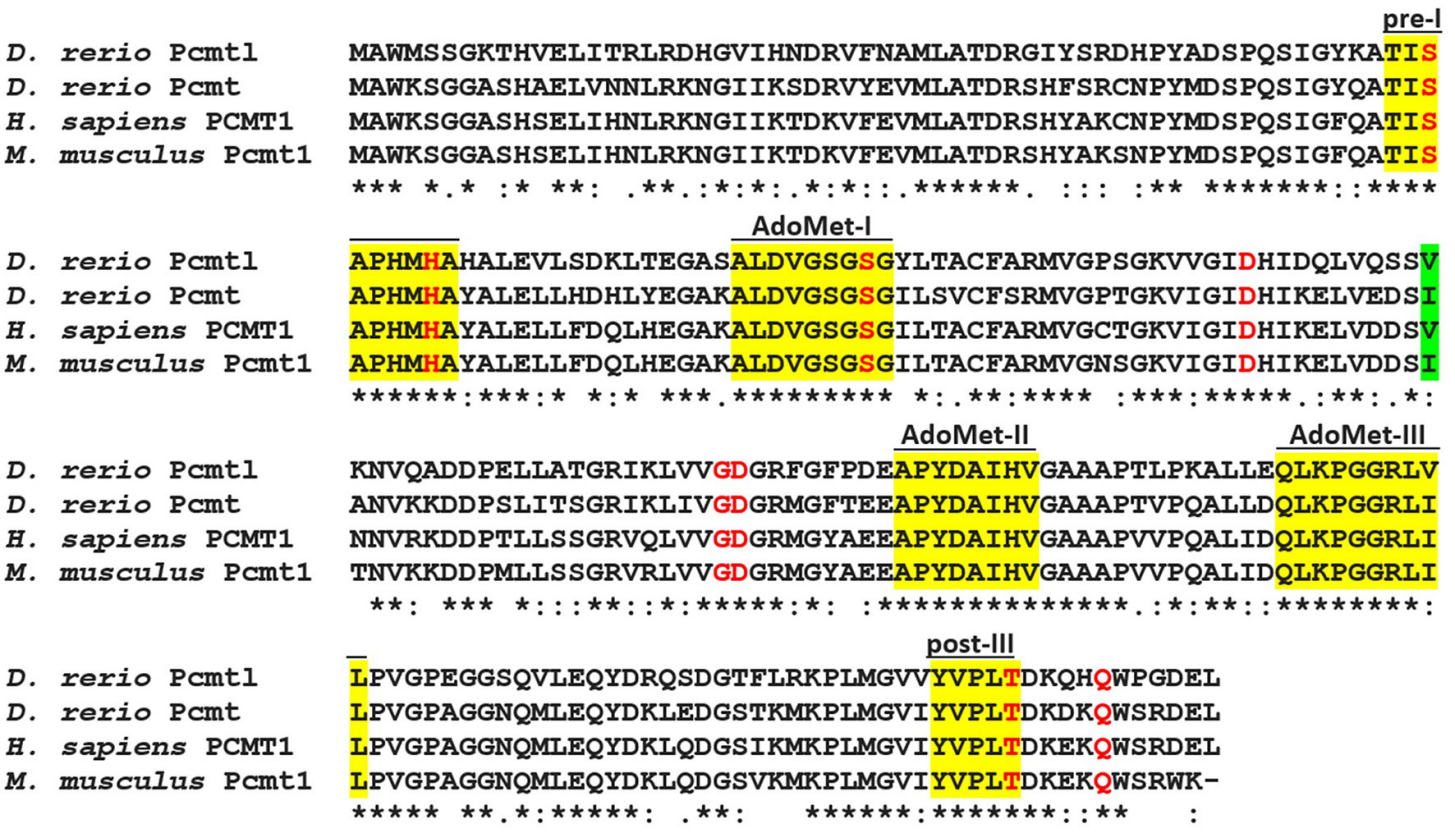
Sequence alignment of human PCMT1, mouse Pcmt1, and the zebrafish homologs Pcmt and Pcmtl. The sequences were aligned with the CLUSTAL Omega software (version 1.2.4) (Sievers et al., 2011) using default settings. Conserved methyltransferase domains are highlighted in yellow [AdoMet I–III, *S*-adenosyl-L-methionine-binding domains; pre-I and post-III, isoaspartyl methyltransferase-specific domains (Kagan et al., 1997a)]. Residues in red were found to directly interact with S-adenosylhomocysteine from crystallography data (Smith et al., 2002). The human polymorphic Ile120Val site is highlighted in green; the zebrafish Pcmt and Pcmtl sequences show an Ile to Val substitution in this same position. (*) Fully conserved residues, (:) residues with strongly similar properties, (.) residues with weakly similar properties. Sequence accession numbers: zebrafish Pcmt, NP_571540.1; zebrafish Pcmtl, NP_957062.1; human PCMT1, NP_001347385.1; mouse Pcmt1, NP_032812.2 (residues 59–283).

The zebrafish genome contains two genes, *pcmt* and *pcmtl*, predicted to encode l-isoaspartyl methyltransferases based on the high sequence similarity with established isoaspartyl methyltransferases. *Pcmt* and *pcmtl* are probable paralogs resulting from an ancient genome duplication event in teleost fish (Taylor et al., 2001). The expression products of the *pcmt* and *pcmtl* genes share 85% and 69% amino acid sequence identity, respectively, with the human PCMT1 protein. The three SAM-dependent methyltransferase domains, AdoMet I – III, and the isoaspartyl methyltransferase domains pre-I and post-III (Kagan and Clarke, 1994; Kagan et al., 1997a) are well-conserved in both Pcmt and Pcmtl, as well as amino acid residues found to directly interact with *S*-adenosylhomocysteine from crystal structure data (PDB: 1I1N) (Smith et al., 2002) (Figure 1). The zebrafish genome contains two additional genes encoding isoaspartyl methyltransferase related proteins (*pcmtd1* and *pcmtd2a*). These “isoaspartyl methyltransferase domain-containing” proteins are also conserved in humans and share approximately 25% sequence identity with the canonical isoaspartyl methyltransferase protein PCMT1. The function of the isoaspartyl methyltransferase domain-containing proteins and the substrates for their putative methyltransferase activity remain currently unknown. Given the much lower sequence conservation between zebrafish Pcmtd1 and Pcmtd2a and established isoaspartyl methyltransferase proteins as compared to the one featured by the zebrafish Pcmt and Pcmtl proteins, only the latter were analyzed further in this study.

**Figure 2:**
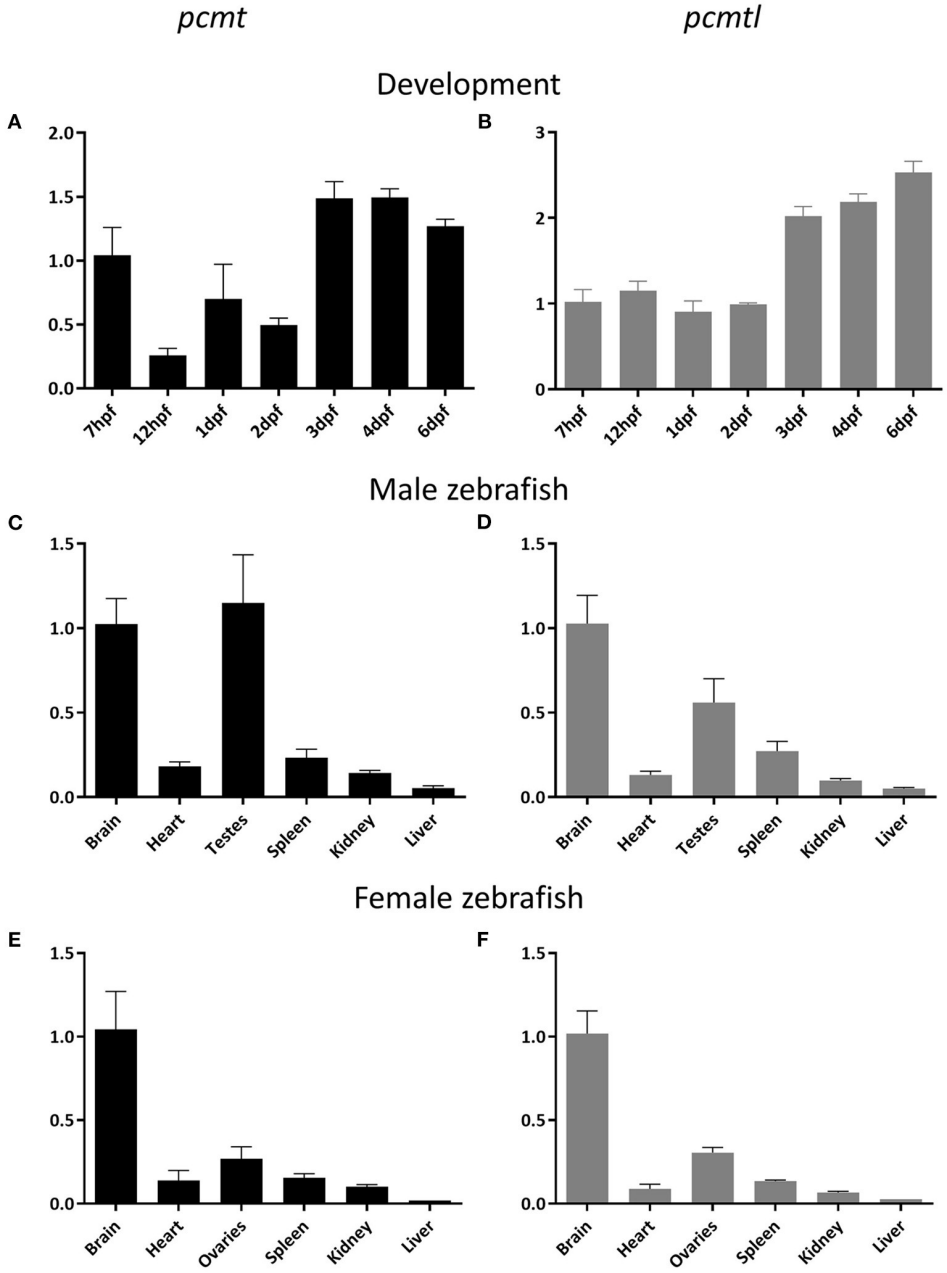
Developmental expression and adult tissue distribution of the *pcmt* and *pcmtl* transcripts in zebrafish. Total RNA was extracted from embryos (*n* = 3) at the indicated developmental stages **(A,B)** and from organs (*n* = 3) of 2-year old male **(C,D)** and female **(E,F)** zebrafish for quantification of the relative *pcmt* (black bars) and *pcmtl* (gray bars) transcript levels using qPCR. Relative expression levels (*eef1a1l1* was used as reference gene) have been normalized to the expression at 7 hpf **(A,B)** or in the brain **(C–F)**. Data shown are means ± SEMs for the 3 biological replicates.

To study *pcmt* and *pcmtl* gene expression profiles in zebrafish, we used a qPCR approach to quantify both transcripts at different developmental stages. We detected *pcmt* and *pcmtl* transcripts in zebrafish embryos as early as 7 h post-fertilization (hpf) and in larvae, up to the last measuring point, at 4 days post-fertilization (dpf) (Figures 2A,B). Transcript levels for *pcmtl* were stable for the first 2 days and then doubled at the 3rd day, while the *pcmt* transcript levels showed more variations during the first 2 days and then also started to stabilize at an overall higher level.

The dynamics observed for *pcmt* expression, where the transcript levels decreased at 12 hpf and increased again at 24 hpf, were intriguing. One possible explanation for the early drop in transcript level would be the presence of a high proportion of maternally transmitted mRNA at the first time point, which then decreases while the endogenous transcription machinery is taking over. To test this hypothesis, we collected eggs directly after mating (30 min post-fertilization) for RNA extraction. Agarose gel analysis of PCR amplicons from egg cDNA showed the presence of both *pcmt* and *pcmtl* transcripts in the eggs confirming the transmission of maternal mRNA for both genes (Supplementary Figure 1). Taken together, our results show that *pcmt* and *pcmtl* transcripts are maternally transmitted and that both genes are endogenously expressed during early stages of development, suggesting that both proteins are needed during early zebrafish development.

### Tissue Distribution of *pcmt* and *pcmtl* in Zebrafish

We next investigated the tissue distribution of both genes at early developmental stages and in adult animals. Previous studies, using *in situ* hybridization, showed that at embryonic and larval stages, *pcmtl* is not spatially restricted to a specific tissue (Thisse and Thisse, 2004). As a main interest of ours was to use the zebrafish model to further elucidate the physiological function of isoaspartyl methyltransferase in the brain, we first performed *pcmt* and *pcmtl* transcript quantification by qPCR on cDNA prepared from pooled head and trunk samples dissected from 7 dpf larvae. We found *pcmt* and *pcmtl* expression in both locations, but we measured 2- to 3-fold higher transcript levels in the head region than the rest of the body (Supplementary Figure 2). We also performed qPCR experiments on cDNAs from different organs of adult (2 year-old) fish. Independently of the gender, a similar pattern was observed for both genes, with transcript detection in all tissues tested, but with highest expression levels found in the brain and testes/ovaries (Figures 2C–F). These results are in good agreement with the tissue distribution previously described for *Pcmt1* in mice (Kim E. et al., 1997; Lowenson et al., 2001; Mizobuchi et al., 2008).

### Enzymatic Properties of the Zebrafish Pcmt and Pcmtl Proteins

For the enzymatic characterization of zebrafish Pcmt and Pcmtl, we recombinantly expressed both enzymes in *E. coli* with an N-terminal His-tag and purified them by nickel affinity chromatography. Virtually homogenous protein preparations were obtained after the single step purification as shown by SDS-PAGE analysis using Coomassie blue staining (Supplementary Figure 3). The isoaspartyl methyltransferase activity of the purified recombinant Pcmt and Pcmtl proteins was characterized via a methanol vapor diffusion assay, using an isoaspartyl containing peptide as substrate and tritiated SAM (Patananan et al., 2014). These assays confirmed that Pcmt and Pcmtl act as isoaspartyl methyltransferases with K_M_ values of 4.2 (± 0.5) μM and 3.6 (± 0.5) μM, respectively, for the methyl acceptor substrate and V_max_ values of 9.1 (± 0.3) and 14.1 (± 0.5) nmol·min^−1^·mg^−1^ protein (the values given are means ± SEMs calculated based on 3 independent saturation curves). The K_M_ values are intermediate compared to isoaspartyl methyltransferase enzymes from other species tested with the same peptide substrate (Table 1). The substrate affinity of the zebrafish enzymes is higher compared to Pcmt from *Arabidopsis thaliana* (K_M_ = 80 μM) and lower compared to PCMT1 from *H. sapiens* (K_M_ = 0.52 μM) (Lowenson and Clarke, 1991; Thapar and Clarke, 2000). Based on our gene expression and enzymatic analyses, the zebrafish Pcmt and Pcmtl enzymes seem to display very similar properties, and the reason for the maintenance of two genes apparently encoding the same function in this organism remains to be elucidated. In practical terms, this meant, however, that both genes had to be targeted in zebrafish to achieve efficient knockdown of isoaspartyl methyltransferase activity for subsequent phenotypic analyses.

**Table 1:** Affinity of isoaspartyl methyltransferase from different species for the peptide substrate KASAisoDLAKY.

**Species**	***K_***M***_* (μM)**	**References**
*A. thaliana* (PIMT1)	80 ± 18	Thapar and Clarke, 2000
*C. elegans*	9.12	Kagan and Clarke, 1995
*D. rerio* (Pcmt)	4.2 ± 0.51	This work
*D. rerio* (Pcmtl)	3.6 ± 0.48	This work
*E. coli*	50.6	Fu et al., 1991
*H. sapiens*	0.52 ± 0.08	Lowenson and Clarke, 1991
Wheat germ	12.7	Mudgett and Clarke, 1993
*T. maritima*	2.8 ± 0.24	Ichikawa and Clarke, 1998

*The values shown are means ± SDs (or SEMs) if available*.

### Transient Knockdown of *pcmt* and *pcmtl* Expression Leads to Lower Isoaspartyl Methyltransferase Activity and Higher Isoaspartyl Levels

**Figure 3:**
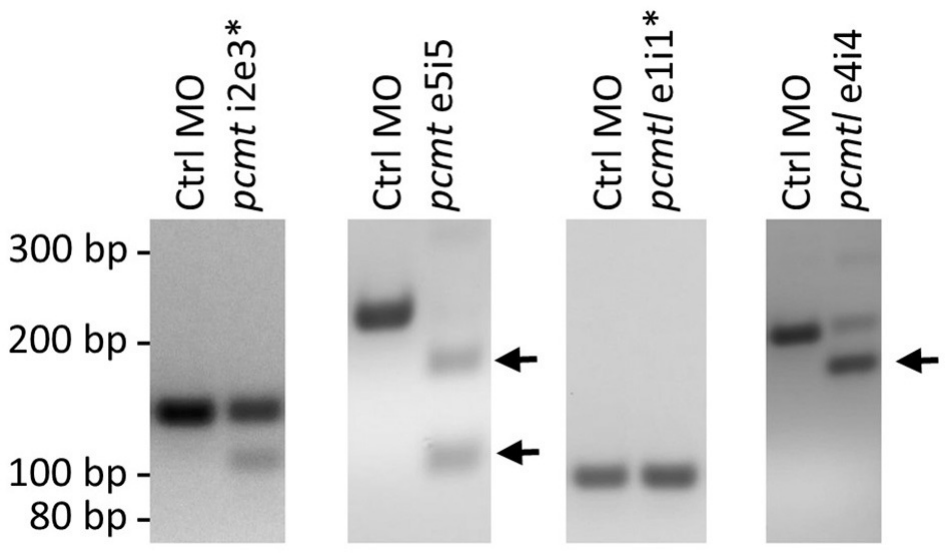
*pcmt* and *pcmtl* morpholino-mediated knockdown efficiency. Splice-blocking MOs (5 ng) targeting *pcmt* (*pcmt*-i2e3 and *pcmt*-e5i5) or *pcmtl* (*pcmtl*-e1i1 and *pcmtl*-e4i4) or a non-targeting control MO (Ctrl MO) were microinjected into zebrafish embryos (1-cell stage) and total RNA was extracted at 1 dpf for cDNA synthesis and PCR amplification using primers spanning the targeted intron-exon boundaries. Agarose gel analysis showed very low efficiency for the *pcmt*-i2e3 and *pcmtl*-e1i1 MOs (asterisks), but high efficiency for the *pcmt*-e5i5 and *pcmtl*-e4i4 MOs leading to exon skipping as indicated by the smaller amplicon sizes (arrows).

To knock down *pcmt* and *pcmtl*, we tested four splice blocking MOs (*pcmt* i2e3, *pcmt* e5i5, *pcmtl* e1i1, and *pcmtl* e4i4). The *pcmt* e5i5 and *pcmtl* e4i4 MOs seemed to exert the highest knockdown efficiency, based on fading or complete disappearance of wild-type amplicons and appearance of shorter amplicons obtained with PCR primers spanning the targeted gene regions (Figure 3); hence only those MOs were used for further experiments. Sequencing of the shorter amplicons revealed partial or complete skipping of exon 5 in *pcmt* e5i5-injected larvae and complete skipping of exon 4 in *pcmtl* e4i4-injected larvae (Supplementary Figure 4). Titration of the amount of each MO to be delivered showed no severe morphological changes following injection of up to 7 ng of each MO when injected individually. Hence, we used co-injection of 3.5 ng of each MO (from now on referred to as *pcmt/l* MO treatment), previously tested to be efficient at transcript level, as a presumably minimally toxic working amount for simultaneously knocking down both genes. In this combined way, the MO treatment led to morphological changes that are described in more details below.

**Figure 4:**
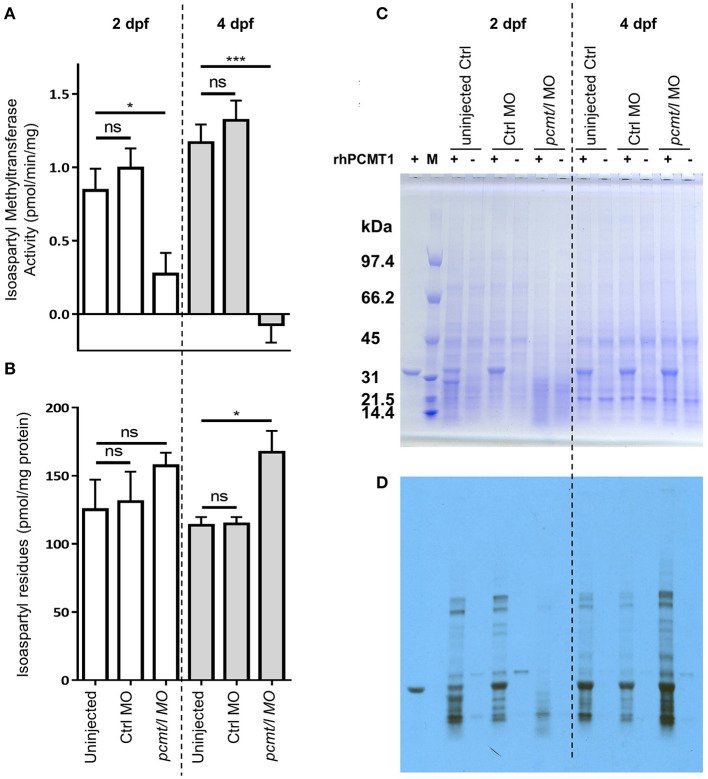
Knockdown of *pcmt* and *pcmtl* leads to lower isoaspartyl methyltransferase activity and higher isoaspartyl levels in zebrafish larvae. Proteins were extracted from uninjected, control MO-injected and *pcmt/l* morpholino-injected larvae and isoaspartyl methyltransferase activity **(A)**, as well as isoaspartyl levels **(B)**, were assayed using the methanol vapor diffusion assay. The protein extracts were also analyzed after labeling in the presence of recombinant human PCMT1 (rhPCMT1) and tritiated SAM by SDS-PAGE **(C)** followed by fluorography **(D)**. The values shown in panels **(A)** and **(B)** are means ± SEMs of 4 biological replicates (ns, not significant; *p-value < 0.05; ***p-value < 0.001). For the SDS-PAGE and fluorography analyses, one representative experiment out of 4 biological replicates is shown. M, molecular weight markers.

In protein extracts prepared from *pcmt/l* morphant larvae showing moderate to normal morphological phenotype, we determined endogenous isoaspartyl methyltransferase activity levels using the same isoaspartyl-containing peptide substrate as for characterization of recombinant zebrafish Pcmt and Pcmtl. In extracts from 2dpf *pcmt/l* morphant larvae, we measured a 72% reduction in the endogenous isoaspartyl methyltransferase activity (compared to extracts from age-matched uninjected or control MO injected larvae) and in extracts from 4 dpf morphant larvae, we did not detect any residual activity (Figure 4A). We next quantified the amount of isoaspartyl residues contained in the zebrafish protein extracts using an assay based on the use of recombinant human PCMT. At 2 dpf, we measured only slightly (not statistically significant) increased isoaspartyl levels in *pcmt/l* morphant protein extracts, which could be due to the residual methyltransferase activity detected at this stage (Figure 4B). At 4 dpf, a statistically significant 1.5-fold increase in isoaspartyl residues was measured in the *pcmt/l* morphants compared to controls.

In addition to measuring the bulk levels of isoaspartyl residues in zebrafish protein extracts, we performed fluorography experiments to determine whether the detected isoaspartyl residues were uniformly distributed among proteins of all sizes or if they were found more specifically in certain proteins only. Protein extracts from zebrafish larvae at 2 and 4 dpf (uninjected, control MO-injected or *pcmt/l* MO-injected) were labeled with [^3^H]SAM using recombinant human PCMT1 enzyme, resolved by SDS-PAGE, and the protein gels analyzed by fluorography for detection of labeled (isoaspartyl-containing) protein bands as described previously (Patananan et al., 2014).

Most striking differences were observed for isoaspartyl labeling intensity and pattern between control MO- and *pcmt/l* MO-injected larvae (Figure 4D; Supplementary Figures 5C,D). Some differences in labeling intensity were also observed between uninjected control and control MO-injected larvae, indicating that, for reasons that remain elusive to us, the MO injection procedure may affect the rate of isoaspartyl formation in proteins to a certain extent (not giving rise, however, to significant changes in bulk isoaspartyl levels as can be seen in Figure 4B). Interestingly, at 2 dpf, Coomassie blue staining consistently showed decreased intensity for all proteins above 31 kDa in the *pcmt/l* morphant protein extracts, despite loading the same amount of protein (based on Bradford assay) as for uninjected and control MO-injected larvae extracts (Figure 4C; Supplementary Figures 5A,B). Similarly, fluorography staining showed lower levels of isoaspartyl residues in these extracts (Figure 4D; Supplementary Figures 5C,D), despite the detection of equal or even slightly higher bulk levels of isomerized residues in the *pcmt/l* morphant extracts using the vapor diffusion assay (Figure 4B). This suggested that the lower labeling intensity in the fluorograph for the *pcmt/l* morphants does not reflect overall lower accumulation of isoaspartyl residues in their proteins, but higher rates of proteolysis with formation of small labeled peptides that are not detected by fluorography, but are well measurable in the isoaspartyl assay in total protein extracts. As we used protease inhibitors during protein extraction, this increased proteolysis presumably took place *in vivo* and not in the protein extracts themselves. Indeed, isoaspartyl accumulation and PCMT downregulation was reported to activate protease activity (Szymanska et al., 1998; Dai et al., 2013; Dho et al., 2013). In stark contrast, nothing points at this increased proteolysis in extracts derived from *pcmt/l* morphant larvae at 4 dpf (equal Coomassie blue staining intensities in all the lanes for 4 dpf samples, as expected given equal loading amounts for all the samples to be compared) (Figure 4C). In addition, fluorography confirmed the increase in the isoaspartyl levels in the *pcmt/l* morphant larvae at 4 dpf (Figure 4D) observed also during isoaspartyl assays in total protein extracts, but seemed not to be associated with some specific proteins only (increased labeling was distributed over the range of protein sizes analyzed).

Taken together, these results confirm the efficiency of the combined *pcmt/l* MO knockdown strategy, with a profound downregulation of the isoaspartyl methyltransferase activity and higher accumulation of isoaspartyl residues in *pcmt/l* morphant larvae. Both effects were more pronounced at 4 dpf than at 2 dpf larvae. It is noteworthy that in the *Pcmt1* knockout mice, isoaspartyl levels are also positively correlated with age (Kim E. et al., 1997). To our knowledge, isoaspartyl level measurements have not been reported for mice at developmental stages, which would correspond to the early stages of life investigated here in zebrafish.

### Morphological Changes Induced Upon *pcmt* and *pcmtl* Knockdown

**Figure 5:**
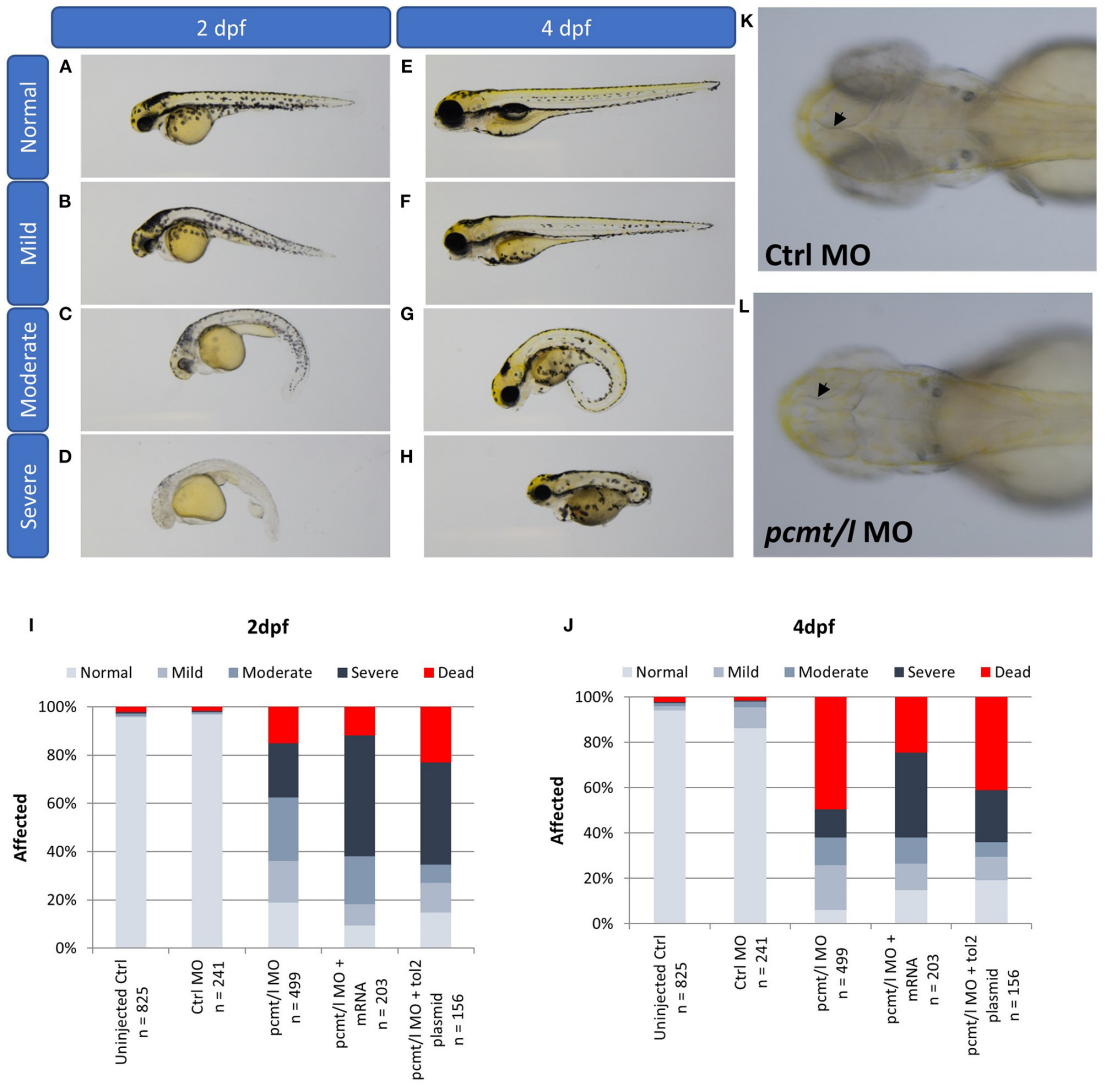
Knockdown of *pcmt* and *pcmtl* leads to morphological changes and developmental delay. The morphological changes were ranked from mild to severe in 2 dpf larvae [**(A–D)**; trunk curvature and decreased pigmentation) and in 4 dpf larvae **(E–H)**; lack of the swim bladder, body curvature, and strong malformation]. Relative quantification of these phenotypes was performed by observation of the indicated numbers of *pcmt/l* morphants compared to control and rescue larvae (exogenous expression of *pcmt/l* via mRNA or *Tol2* transgenesis in the morphant background) at 2 dpf **(I)** and 4 dpf **(J)**. Arrows indicate the internal brain structure showing smaller brain size in the *pcmt/l* morphant larvae **(L)** compared to control MO-injected larvae **(K)**.

Assessment of morphological changes in *pcmt*/*l* morphant larvae revealed various phenotypes at 2 and 4 dpf (Figure 5). We ranked the intensity of the morphological changes from mild to severe (Figures 5A–H). At 2 dpf, a slight body curvature was ranked as a mild phenotype, a curved trunk and small body size as a moderate phenotype, and decreased melanization and a strong malformation as a severe phenotype. At 4 dpf, lack of swim bladder, curved trunk, and strong malformation were ranked as mild, moderate, and severe phenotypes, respectively. Quantification of these phenotypes highlighted a substantial increase in abnormal morphological changes in *pcmt/l* morphants as compared to uninjected and control MO-injected embryos (Figures 5I,J). At 2 dpf, 15% of the *pcmt*/*l* morphant larvae were dead and 22, 26, and 17% showed severe, moderate, and mild dysmorphologies, respectively; 19% were normal. The deleterious effects became even more pronounced at 4 dpf where about 50% of the *pcmt/l* morphant larvae were dead. In contrast, more than 95 and 85% of embryos injected with control MO (as well as uninjected embryos) showed a normal phenotype at 2 and 4 dpf, respectively.

Another interesting observation in the *pcmt/l* morphant larvae was the small head size (Figure 5L). We noticed a general developmental delay and smaller body size in *pcmt/l* morphants, which seemed consistent with the smaller body size observed for *Pcmt1* knockout mice (Yamamoto et al., 1998). However, the mice exhibited increased brain size in contrast with the smaller brains observed here in *pcmt/l* morphant larvae. It may be speculated that the increased brain size in case of the *Pcmt1* knockout mice could be preceded at embryonic stages by smaller brains, leading to activation of growth signaling pathways. In general, it should be noted though that a direct comparison between the zebrafish model and the mouse model is not possible, since our observations in zebrafish were made in the first 4 days of life while body and brain size measurements in mice were reported for an age of 4–7 weeks (Yamamoto et al., 1998; Farrar C. et al., 2005).

In order to evaluate whether these morphological phenotypes were specifically caused by *pcmt/l* deficiency, we prepared mRNA transcripts and recombinant plasmids to restore expression of both genes in the *pcmt/l* morphants. Based on preliminary titration assays to determine maximum tolerated amounts, we injected 100 pg of mRNA transcripts or 20 pg of plasmid DNA along with the *pcmt/l* MOs to perform the rescue experiments. For the 2 dpf larvae, neither the mRNA transcripts nor the expression plasmids encoding *pcmt* and *pcmtl* showed a rescue effect; at the contrary, they even amplified the severity of the dysmorphology. By contrast, for the 4 dpf larvae, both rescue strategies reversed, although only very partially, the deleterious effects in *pcmt/l* morphants, with a higher proportion of normal larvae and a lower proportion of dead larvae. The most straightforward explanation for these observations would be that most (4 dpf), if not all (2 dpf) of the dysmorphology phenotype is caused by non-gene specific, off-target effects of the *pcmt/l* MOs. This explanation seems, however, unlikely, given the very modest (control-like) effects at morphological level that were observed when injecting the *pcmt* and *pcmtl* MOs separately, as reported above. It is also important to note that any maternally transmitted *pcmt* and *pcmtl* mRNAs would not be affected by the MOs used in this study (as we used splice-blocking and not translation-blocking MOs). Maternally transmitted mRNAs could explain the less pronounced morphological defects observed in 2 dpf vs. 4 dpf *pcmt/l* morphants. In addition, the presence of these maternal mRNAs could lead to toxicity (rather than rescue effects) upon injection of our rescue transcripts or plasmids at 2 dpf, due to overexpression of the target proteins.

### Interplay Between PCMT and Calcium Signaling in Zebrafish Brain

To investigate potential disturbance of brain function induced by *pcmt* knockdown and given substantial preliminary evidence indicating a role of PCMT in calcium signaling, we used the *beta-actin*:GCaMP6f transgenic zebrafish line (Sieger et al., 2012) to monitor cytoplasmic calcium levels in the brain of zebrafish larvae in the context of *pcmt/l* deficiency. We injected 1-cell stage *beta-actin*:GCaMP6f eggs with either control or *pcmt/l* MOs. At 4 dpf, the larvae were exposed to the proconvulsant drug pentylenetetrazol (PTZ) to induce calcium release (Tao et al., 2011) and resulting fluorescence signals were imaged. It should be noted that only larvae with no or only moderate morphological changes were chosen to perform this experiment.

**Figure 6:**
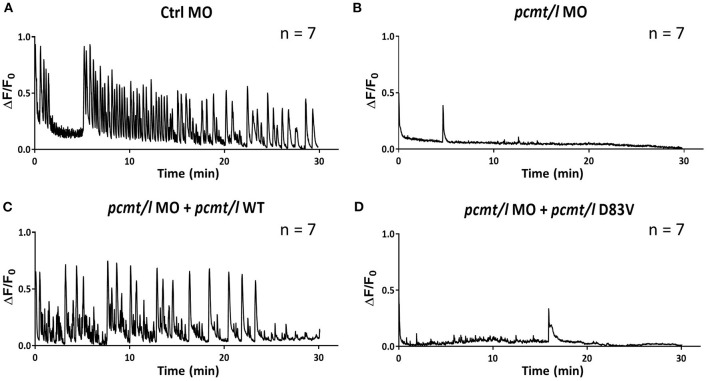
Knockdown of *pcmt* and *pcmtl* leads to disruption of calcium fluxes in the brain of zebrafish larvae. Calcium fluxes in response to PTZ exposure were imaged by fluorescence microscopy in the brain region of *beta-actin*:GCaMP6f larvae injected with control MO **(A)**, *pcmt/l* MOs **(B)**, and with co-injection of *pcmt/l* MOs and a plasmid for wild-type Pcmt and Pcmtl **(C)** or of *pcmt/l* MOs and a plasmid for catalytically inactive Pcmt and Pcmtl (D83V) **(D)**. The 0 min time point coincides with the start of calcium imaging after a 30-min pre-incubation with PTZ. The traces shown are representative for 7 independent replicates.

As shown by the oscillations of the fluorescence signal in the brain of larvae injected with control MO, PTZ induced large calcium transients over the entire duration of the experiment (Figure 6A). Interestingly, *pcmt/l* morphant larvae showed no major calcium peaks during the time-lapse imaging (Figure 6B), indicating strongly decreased transient calcium fluxes into the cytoplasm of the brain cells of these larvae. In order to confirm the gene specificity of this effect, we performed rescue experiments in which we injected *beta-actin*:GCaMP6f eggs with mixtures of *pcmt/l* MOs and pGEMT expression plasmids for wild-type *pcmt* and *pcmtl* or for catalytic null mutants (D83V mutant *pcmt* and *pcmtl*) lacking isoaspartyl methyltransferase activity (Cimmino et al., 2008). Clearly, restoring the expression of wild-type *pcmt* and *pcmtl* (Figure 6C), but not of the catalytically inactive proteins (Figure 6D), rescued the calcium transients induced by PTZ. These observations suggest that the strong calcium signaling defect observed in the *pcmt/l* morphant larvae is specifically caused by down-regulation of *pcmt* and *pcmtl* expression and moreover, that this effect is dependent on the methyltransferase activity of the enzymes. The clear rescue effect observed for the calcium phenotype contrasts with the much more partial recovery of the morphological defects observed in 4 dpf *pcmt/l* morphants co-injected with *pcmt* and *pcmtl* expression plasmids. This indicates a much higher gene specificity for the calcium phenotype compared to the observed morphological abnormalities in the *pcmt/l* morphants.

### *Pcmt1* Knockout in Mouse HT22 Cells

**Figure 7:**
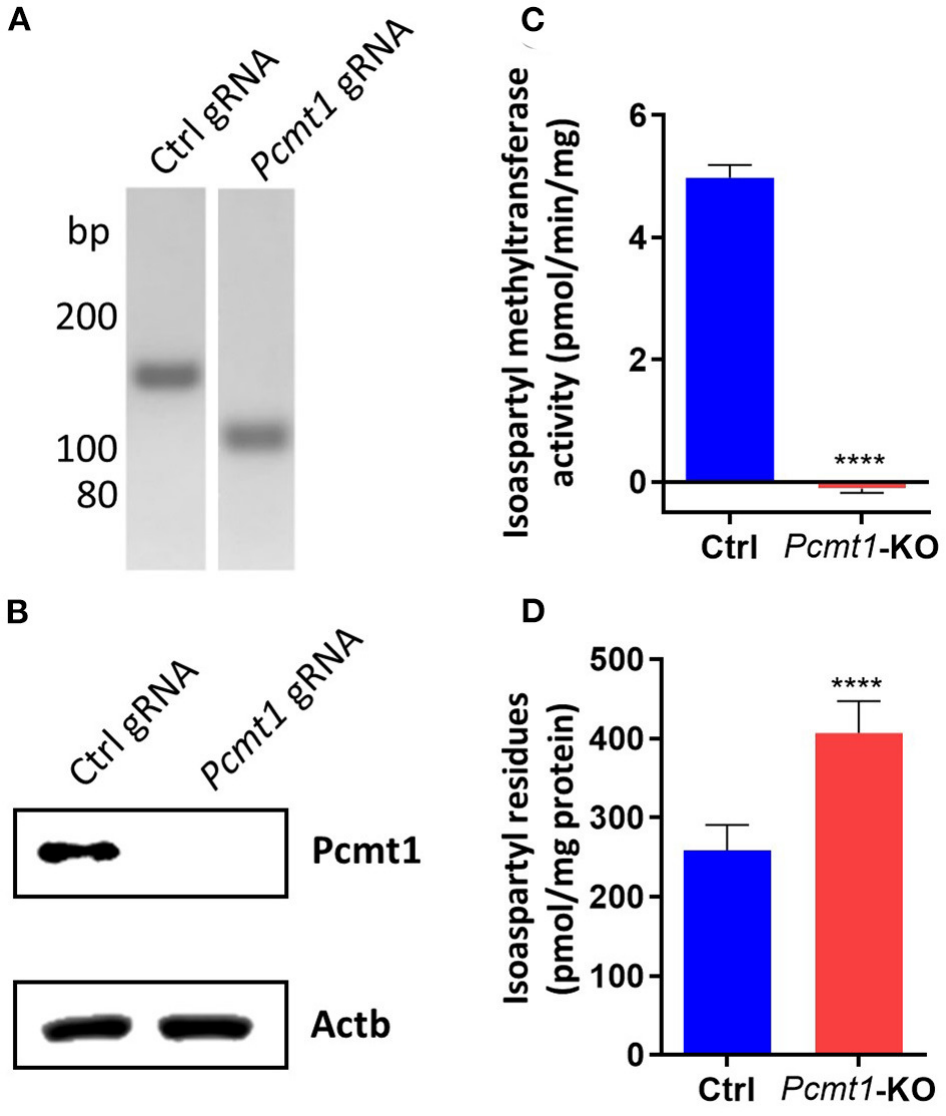
*Pcmt1* knockout in mouse hippocampal HT22 cells by CRISPR/Cas9 technology. Confirmation of the expected 40-bp deletion in the HT22 *Pcmt1* KO cell line by PCR amplification of the *Pcmt1* target site from genomic DNA **(A)**. The generated KO cell line was also validated by Western blotting using primary antibodies against the Pcmt1 protein **(B)**, isoaspartyl methyltransferase activity assay **(C)**, and measurement of isoaspartyl levels in crude protein extracts **(D)**. The values shown are means ± SEMs of 6 biological replicates (****p < 0.0001).

To test whether the effect of PCMT on calcium fluxes is conserved, we knocked out *Pcmt1* in HT22 cells (Liu et al., 2009), a mouse hippocampal-derived cell line, using CRISPR/Cas9 technology. The *Pcmt1* KO cell line was validated by PCR analysis (confirming the expected 40-bp deletion, leading to an early stop codon) and Western blotting, with no residual Pcmt1 protein detected (Figures 7A,B). Consistent with this, no isoaspartyl methyltransferase activity was detected in the *Pcmt1* knockout cells (Figure 7C). Moreover, we found a 1.6-fold increase of isoaspartyl levels in protein extracts prepared from *Pcmt1* KO cells compared to control extracts (Figure 7D). This increase was more moderate than the one previously reported in tissue extracts of *Pcmt1* knockout mice (4- to 8-fold higher isoaspartyl levels than in control extracts) (Kim E. et al., 1997), but in the same study it was shown that this accumulation was age-dependent, with a 2.7-fold increase of isoaspartyl levels in protein extracts from red blood cells taken from *Pcmt1* knockout mice at the age of 24 or 59 days (Kim E. et al., 1997).

### Calcium Signaling in HT22 *Pcmt1* Knockout Cells

**Figure 8:**
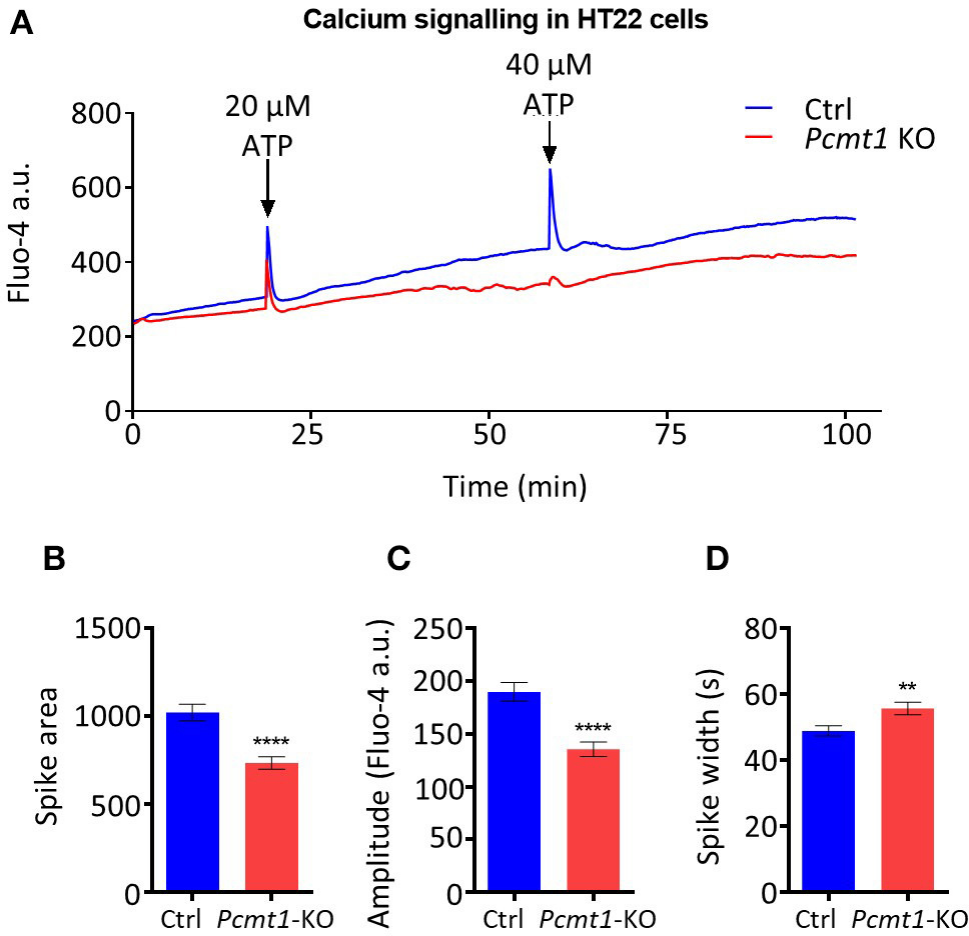
Impaired calcium response in *Pcmt1* knockout HT22 cells. Control or *Pcmt1* KO HT22 cells, stained with Fluo-4 AM, were exposed to 20 μM ATP, followed by a second stimulation with 40 μM ATP at the indicated times, and imaged by fluorescence microscopy in a controlled environment **(A)**. The calcium spike observed after the first ATP stimulation was analyzed for peak area **(B)**, peak amplitude **(C)**, and peak width **(D)**. The fluorescence traces shown are representative of 3 imaged replicate wells. The values shown are means ± SEMs of signals extracted from 150 cells (**p-value < 0.001; ****p-value < 0.0001).

We then used the generated *Pcmt1* knockout cell line to monitor cytosolic calcium fluxes following ATP stimulation, using the fluorescent calcium indicator Fluo-4. We found that both control and *Pcmt1* knockout cells responded to the first ATP stimulation by a spike in cytosolic calcium concentration while upon the second ATP stimulation only the control cells responded with a similar calcium transient (Figure 8A). By comparing the first spike between control and *Pcmt1* knockout cells, we observed a smaller peak area with a lower amplitude but with a larger width in the knockout cells (Figures 8B–D). The lower peak area and amplitude indicate lower calcium concentration. In addition, the larger peak width indicates a slower decrease in the intracellular calcium concentration back to basal levels. This delay suggests a lower buffering capacity in the knockout cells, which could be due to a lower binding capacity or affinity of calcium binding proteins such as calmodulin or defects in ion pumps, such as SERCAs or PMCAs, involved in organellar calcium reuptake or extracellular calcium release, respectively. These results were reminiscent of the observations in the *pcmt/l* morphant zebrafish larvae in that they point toward PCMT being important to sustain cellular calcium homeostasis.

## Discussion

### Zebrafish Expresses Two Isoaspartyl Methyltransferases

Gene duplication is a powerful tool for the acquisition of new function. In 1970, Susumu Ohno presented a now classical view of the evolution of duplicated genes: whereas one gene must conserve the function, the second one may acquire mutations, either in the coding or non-coding regions, which have the potential to lead to dosage compensation, sub- or neo-functionalization (Conant and Wolfe, 2008). The zebrafish genome encodes two proteins (Pcmt and Pcmtl) predicted to catalyze the same reaction as PCMT1, based on the high amino acid sequence conservation. Here we confirm this prediction experimentally, demonstrating isoaspartyl methyltransferase activity (transfer of a methyl group from the cofactor SAM to the isoaspartyl residue) for both purified recombinant Pcmt and Pcmtl. For the *in vitro* activity assay, we used the non-apeptide KASAisoDLAKY, a high affinity substrate for PCMT1 used before to characterize the activity of PCMT homologs from many different species (Ichikawa and Clarke, 1998).

All amino acid residues of the three AdoMet domains and the two isoaspartyl methyltransferase domains are strictly conserved between Pcmt and Pcmtl except for Ile180 in the AdoMet-III domain, which is replaced by a Val in Pcmtl. In the human PCMT1 protein, the Ile120Val polymorphism has been shown to affect the enzymatic properties, with lower activity for the Val120 form (Rutherford and Daggett, 2009). For the zebrafish proteins (Ile120 for Pcmt and Val120 for Pcmtl), we found a slightly higher affinity, but also an increased activity for the protein containing a Val at this position (Pcmtl). Compared to PCMT homologs from other species, the substrate affinity of both zebrafish enzymes can be classified as intermediate among homologs with higher affinity, like human PCMT1, and lower affinity, like *C. elegans* PCM-1 and plant PCMTs, based on the reported K_M_ values (Table 1). The almost 10-fold lower substrate affinity of the zebrafish proteins compared to human PCMT1 cannot be attributed to differences in the AdoMet or isoaspartyl methyltransferase domains, since those are strictly conserved between the proteins. Even more striking, the affinity of human PCMT1 for KASAisoDLAKY is 160-fold higher than the one of *A. thaliana* PCMT although the AdoMet and isoaspartyl methyltransferase domains are also strictly conserved between those two proteins. Thus, it appears that the affinity for the isoaspartyl peptide substrate is highly influenced by amino acid residues outside of those conserved domains. Also for other peptide and protein substrates, the affinity of isoaspartyl methyltransferase has been shown previously to vary largely between species and it was suggested that the enzyme properties may have undergone evolutionary adaptation to the type of damaged protein substrates encountered *in vivo* (Thapar and Clarke, 2000).

Our results showed that both *pcmt* and *pcmtl* transcripts are maternally transmitted in zebrafish, as we were able to detect both transcripts in eggs collected within 30 min after mating. The transcript levels continued to increase during the first few days of embryonic development. As isoaspartyl levels have previously been shown to increase with age in mice (Kim E. et al., 1997; Lowenson et al., 2001), such an early expression of the repair enzyme during development could indicate an alternative function, for instance a regulatory function via methylation of specific proteins as opposed to the more global housekeeping protein repair role that might become more important with aging. In rats, the *Pcmt* transcript levels also increased in the brain from embryonic day 15 (E15) up to 28-week-old (28W) (Shirasawa et al., 1995). The existence of a conserved role for Pcmt during embryonic development may be further supported by its importance for neuronal differentiation, indicated by the observation that *Pcmt1*-deficient PC12 cells failed to differentiate *in vitro* (Dung et al., 2016), and by the developmental delay and lack of neuronal maturation in *Pcmt1* knockout mice (Farrar C. E. et al., 2005).

Using qRT-PCR, we found that adult zebrafish express *pcmt* and *pcmtl* in all tested tissues, with highest expression levels in the brain and testes or ovaries. A similar expression pattern, based on transcript and isoaspartyl methyltransferase activity levels, was also found in mice (Kim E. et al., 1997; Lowenson et al., 2001). Furthermore, in *Pcmt1* knockout mice, the brain was the most affected organ with the mice dying due to fatal epileptic seizures. Interestingly, these seizures as well as the short lifespan of *Pcmt1* knockout mice were prevented by restoring *Pcmt1* expression specifically in the brain (Kim E. et al., 1997; Lowenson et al., 2001). Taken together, these observations indicate a general support function of PCMT in all tissues of the analyzed species, including now zebrafish, but more critical roles in the brain. The high expression levels of PCMT in testes might be important to preserve fertilization competent sperm for several weeks of storage (Chavous et al., 2000). Similarly, in plant seeds protein homeostasis is essential given long periods of quiescence before the seeds start to germinate. Accordingly, in *A. thaliana*, PCMT has been shown to affect seed longevity and germination vigor and to protect the seeds from heat stress (Ogé et al., 2008). In addition, high PCMT expression in gametes may contribute to the rejuvenation process during gametogenesis through the removal of damaged proteins (Unal et al., 2011).

While all the above helps justify high expression of *pcmt* and *pcmtl* in brain, testes and ovaries, the reason for the conserved expression of two enzymes that catalyze the same reaction, with very similar kinetic parameters and tissue distribution in zebrafish is not clear. However, it is not the only organism in which the expression of two *PCMT* genes has been maintained. *A. thaliana* expresses two isoaspartyl methyltransferase enzymes encoded by two different genes (*PIMT1* and *PIMT2*) and one difference found so far is the subcellular localization, with PIMT2 being targeted to the nucleus (Xu et al., 2004; Villa et al., 2006). Another difference was identified at the level of the regulation of gene expression; *PIMT2* expression is more sensitive to induction by stress or abscisic acid treatment than the constitutively expressed *PIMT1* (Mudgett and Clarke, 1994; Xu et al., 2004). We saw subtle differences in the transcription dynamics of the *pcmt* and *pcmtl* genes in zebrafish during early development, but further investigations on transcriptional regulation and subcellular localization of Pcmt and Pcmtl in zebrafish may lead to a better understanding on their “raison d'être” in this organism. Interestingly, zebrafish Pcmt, but not Pcmtl, possesses an RDEL motif at the C-terminus (GDEL in Pcmtl), suggesting a possible ER localization of this enzyme, as was proposed for some mammalian Pcmt isoforms (MacLaren et al., 1992; Potter et al., 1992; Galus et al., 1994). The zebrafish Pcmt and Pcmtl proteins are small enough to diffuse through the nuclear pores, but TargetP-2.0 (Almagro Armenteros et al., 2019) does not predict signal or mitochondrial targeting peptides for either of the proteins (NCBI Reference Sequences NP_571540.1 and NP_957062.1 used for analysis). However, other protein isoforms with N-terminal extensions may be generated from the *pcmt* and *pcmtl* genes and more experimental work will be needed to determine whether targeting mechanisms to any specific subcellular localisations exist for the zebrafish proteins.

### Zebrafish Model for PCMT Deficiency

The *pcmt/l* morphant larvae showed more than 73% decrease in the isoaspartyl methyltransferase activity at 2 dpf and no detectable activity at 4 dpf. This profound decrease measured for the enzymatic activity in the *pcmt/l* morphants strongly indicates that Pcmt and Pcmtl represent the major protein isoaspartyl methyltransferases in zebrafish. Despite this drastic decrease in isoaspartyl methyltransferase activity in the *pcmt/l* morphant larvae, there was no statistically significant increase in isoaspartyl levels at 2 dpf and only a 1.5-fold increase at 4 dpf. The absence of isoaspartyl accumulation at 2 dpf and the low accumulation at 4 dpf, as compared to an up to 8-fold increase of isoaspartyl levels measured in *Pcmt1* knockout mouse tissues (Kim E. et al., 1997), may be explained in several ways. First, the age difference of the analysis in fish vs. mice [30–40 days old (Kim E. et al., 1997)] may explain part of the discrepancy as isoaspartyl accumulation is known to increase with age. Two or four days of development might not have been enough time for reaching a more robust isoaspartyl accumulation in our zebrafish model, but this time constraint for analysis is imposed by MO technology as knockdown efficiency is transient and starts to dissipate from 5 dpf onwards. Second, knockdown of the isoaspartyl methyltransferase activity might lead to activation of proteolytic pathways limiting the accumulation of isoaspartyl residues in proteins by an alternative mechanism (Patananan et al., 2014). Indeed, SDS-PAGE analysis of protein extracts from 2 dpf *pcmt/l* morphants showed increased proteolysis in those samples and comparison of results obtained by isoaspartyl assay in total protein extracts vs. fluorography supported the idea of increased proteolysis of isoaspartyl-containing proteins specifically induced in the *pcmt/l* morphants. The latter might therefore be an interesting model for further investigations on proteases more specifically acting on isoaspartyl-containing proteins. Third, even though we could not detect isoaspartyl methyltransferase activity anymore in 4 dpf *pcmt/l* morphants, morpholino technology usually does not lead to a complete loss of function, meaning that some residual activity, below the detection limit of our enzymatic assay, may have persisted in the zebrafish morphants, limiting isoaspartyl accumulation. Fourth, despite the relatively low sequence conservation between Pcmt and Pcmtl and the isoaspartyl methyltransferase domain containing proteins Pcmtd1 and Pcmtd2a, we cannot exclude that the latter proteins also play a role in limiting isoaspartyl damage in proteins. Zebrafish may be a nice model to start investigating this possibility in an *in vivo* setting. Finally, the contribution of maternal *pcmt* and *pcmtl* mRNAs, which are not affected by the splice blocking MOs used in this study, may explain lower gene silencing effects at 2 dpf vs. 4 dpf. This could at least partially explain higher residual methyltransferase activity (and lower isoaspartyl damage) as well as less pronounced morphological defects at 2 dpf compared to 4 dpf in the *pcmt/l* morphants.

We observed indeed important morphological abnormalities and increased mortality in *pcmt/l* morphants compared to control larvae. MO treatment of zebrafish embryos can lead, independently from the targeted gene, to dysmorphic phenotypes that cannot be reproduced by gene deletion strategies (Kok et al., 2015). This discrepancy can be explained by off-target effects of the MOs and/or by compensatory mechanisms that are activated in the null mutants, but not in the morphants (Rossi et al., 2015). We believe therefore that gene knockdown using MOs remains a valuable strategy, especially when care is taken, like in this study, to titrate the injected MO amounts down to concentrations that achieve efficient downregulation of the target gene, while minimizing toxic, likely off-target effects. In our titration experiments, we found no severe morphological changes when injecting up to 7 ng of each of the *pcmt* or *pcmtl* MO individually. Based on knockdown efficiency analyses, this treatment leads to profound silencing of each of the genes, but the absence of phenotype is not surprising given the functional redundancy of both genes. In contrast, co-injection of 3.5 ng of each of these MOs led to important increases in lethality and dysmorphology, as described. This indicates that isoaspartyl methyltransferase function critically supports a normal early development in zebrafish even if we cannot exclude contribution of off-target effects, given the only partial recovery of the morphological defects observed in our rescue experiments. As alluded to above, it would be interesting to investigate in the zebrafish model, if Pcmt and/or Pcmtl, beyond their overall protein repair function, may catalyze regulatory posttranslational carboxyl methylation reactions, for example at the C-terminus of signaling proteins involved in developmental pathways.

### Interplay Between PCMT and Calcium

There is substantial evidence in the PCMT literature for an implication of this protein in calcium-related processes. The ubiquitous Ca^2+^-binding protein calmodulin is one of the PCMT substrates that has been discovered early on. This protein accumulates high levels of isoaspartyl residues during *in vitro* as well as *in vivo* aging, with functional consequences (Gagnon et al., 1981; Johnson et al., 1985; Potter et al., 1993). *In vitro* aged, isoaspartyl-containing calmodulin was also degraded faster when injected into *Xenopus laevis* oocytes than native calmodulin (Szymanska et al., 1998). Certain mammalian Pcmt1 isoforms (as well as zebrafish Pcmt) contain a C-terminal RDEL motif (MacLaren et al., 1992; Potter et al., 1992; Galus et al., 1994), an endoplasmic reticulum retention signal, suggesting a possible localization of the enzyme also in this organelle well-known for its important role in regulating intracellular calcium levels. In addition, one of the major substrates for Pcmt1 found through the proteomic analysis of *Pcmt1* knockout mouse brains is calreticulin (Vigneswara et al., 2006), an endoplasmic reticulum resident protein and calcium regulator. Finally, the EF-hand (Asp-X-Asp-X-X-Gly) motif found in a large number of calcium-binding proteins contains two aspartate residues which are prone to isoaspartyl formation affecting calcium binding (Friedberg, 1988).

In order to find evidence for the interplay between PCMT and calcium also in a whole organism model, we knocked down *pcmt* and *pcmtl* in a transgenic zebrafish line that expresses the GCaMP6f protein, a genetically encoded cytosolic calcium indicator whose fluorescence intensity increases upon calcium binding. PTZ is a drug that is widely used as a seizure-inducing agent in animal models, including zebrafish (Baraban et al., 2005), and it was reported to induce calcium release into the cytosol from intracellular pools (Sugaya and Onozuka, 1978). Here we showed that the knockdown of *pcmt/l* in zebrafish drastically suppressed the calcium fluxes induced in the brain in response to PTZ stimulation, a phenotype that was rescued by restoring expression of wild-type, but not of catalytically inactive forms, of Pcmt and Pcmtl. This observation strongly suggested that the isoaspartyl methyltransferase activity plays an important role in supporting calcium movements in the brain. We sought further support for this role in our mouse hippocampal HT22 cell model where we used the Fluo-4 dye for calcium imaging by fluorescence microscopy. Upon stimulation of the cells with ATP, both control and *Pcmt1* knockout cells showed a transient increase in intracellular calcium concentrations. Interestingly, upon a second stimulation with ATP, the control cells again responded with a spike in intracellular calcium concentration, which was not observed in the *Pcmt1* knockout cells. Even though the HT22 knockout cells responded to the first ATP stimulation, the amplitude and dynamics of the response were different than in the control cells, indicating a lower calcium buffering capacity (to recover basal cytosolic calcium concentrations) in the knockout cells.

These observations strongly suggest that isoaspartyl methyltransferase activity plays a conserved role in cellular calcium fluxes and both the zebrafish and HT22 PCMT deficient models will be instrumental in investigating the mechanism underlying this role. Since calmodulin is prone to isoaspartyl damage and since its calcium binding affinity might be affected by the accumulation of isoaspartyl residues (Billingsley et al., 1985), it is tempting to speculate that this protein may mediate at least partially the abnormal calcium responses observed in our PCMT deficient models. Impaired release or re-uptake of calcium from subcellular organelles such as the ER or the mitochondria may also contribute to the observed calcium phenotypes. Accordingly, PCMT1-deficient A549 cells were recently reported to display mitochondrial dysmorphology and decreased intracellular ATP levels (Ogasawara et al., 2016). Compromised energy levels may have led to the virtually absent calcium response to the second ATP stimulation in the HT22 *Pcmt1* knockout cells. Deficient isoaspartyl damage repair could also lead more directly to decreased activity of certain calcium channels and/or signaling proteins that are important for inducing intracellular calcium fluxes in response to extracellular stimuli. The release of calcium from the ER in response to ATP is dependent on phospholipase C (PLC) activity, following activation of GTP-dependent GPCR. PLC catalyzes the hydrolysis of phosphatidylinositol 4,5-bisphosphate (PIP_2_) to diacylglycerol (DAG) and IP_3_, which in turn binds to the IP_3_ receptor (IP_3_R) in the ER membrane leading to the release of calcium (Salter and Hicks, 1995). It is currently not known whether PLC is a substrate for PCMT. Similar to *Pcmt1* knockout mice, however, knockout mice of the β1 isoform of PLC developed epilepsy (Kim D. et al., 1997), indicating that the epileptic seizures in the *Pcmt1* knockout mice may be mechanistically connected to PLC function, although this remains highly speculative at this point.

In summary, our work shows that it is suitable to use zebrafish as an *in vivo* model organism to study the physiological role of isoaspartyl methyltransferase. In particular, using this model, we provide for the first time *in vivo* evidence for a critical role of PCMT function in the cellular calcium response to extracellular stimuli and show that this role seems to be conserved in mammalian cells.

## Data Availability Statement

The original contributions presented in the study are included in the article/Supplementary Material, further inquiries can be directed to the corresponding author.

## Ethics Statement

Ethical review and approval was not required for the animal study because the present study did not involve any procedure within the meaning of Article 3 of the Directive 2010/63/EU and as such it was not subjected to authorization by an ethics committee.

## Author Contributions

RS, MC-M, and CL conceived and designed the experiments and wrote the manuscript. RS, MC-M, TM, MM, J-FC, and RW performed the experiments. RS, MC-M, MM, J-FC, and RW analyzed the data. AS, AC, SC, and CL supervised the experiments. All authors reviewed and approved the manuscript.

## Conflict of Interest

The authors declare that the research was conducted in the absence of any commercial or financial relationships that could be construed as a potential conflict of interest.

## Footnotes

^1^http://www.sanger.ac.uk/htgt/wge/find_crisprs

## Supplementary Material

Supplementary Figure 1 Agarose gel showing PCR amplicons of *pcmt* and *pcmtl* from zebrafish egg and embryo cDNA. Total RNA was extracted from eggs (30 min post-fertilization) and embryos at 12 and 24 hpf for cDNA synthesis. PCR amplification using primers specific for the *pcmt* and *pcmtl* genes confirmed the presence of both transcripts at the 3 tested stages of development. Beta-actin (*actb*) was used as a reference gene.

Supplementary Figure 2 Expression of *pcmt* and *pcmtl* transcripts in the head and trunk regions of zebrafish larvae. The head was separated from the trunk of 7 dpf zebrafish larvae using a scalpel and samples were placed directly on dry ice for RNA extraction. Relative transcript levels of *pcmt* (red bars) and *pcmtl* (blue bars) were determined by qPCR, using *eef1a1l1* as reference gene. Data shown are means ± SEMs for the 3 biological replicates. Each replicate consisted of a pool of 10 head or trunk samples.

Supplementary Figure 3 SDS-PAGE analysis to assess the purity of recombinant zebrafish Pcmt and Pcmtl preparations. Zebrafish Pcmt and Pcmtl were expressed as N-terminal His-tag fusion proteins in *E. coli* (expected MW of 27 kDa for both recombinant proteins) and purified by nickel affinity chromatography followed by dialysis for desalting. For each preparation, the equivalent of 10 μg protein was loaded onto a 12% polyacrylamide gel under denaturing conditions, followed by Coomassie blue staining.

Supplementary Figure 4 Morpholino strategy used for knocking down *pcmt* and *pcmtl* in zebrafish larvae. **(A)** The *pcmt* e5i5 MO leads to two events, whole and partial skipping of exon 5. Complete skipping of exon 5 leads to a frameshift after amino acid 99 and an early stop codon after 132 amino acids. Partial skipping of exon 5 leads to a frameshift after amino acid 122 and an early stop codon after 155 amino acids. In both cases, the predicted truncated proteins miss the AdoMet-II, AdoMet-III and post-III domains that are required for enzyme activity. **(B)** The *pcmtl* e4i4 MO leads to skipping of the whole exon 4 leading to a frameshift after amino acid 53 and an early stop codon after amino acid 58. The resulting truncated Pcmtl protein is missing all the conserved domains that are important for the enzyme activity. Exons and corresponding numbers of nucleotides are represented by the colored boxes. Ensembl transcripts pcmt-201 and pcmtl-201 were taken as reference.

Supplementary Figure 5 Knockdown of *pcmt* and *pcmtl* leads to higher isoaspartyl levels in zebrafish larvae. Protein extracts of uninjected, control MO-injected and *pcmt/l* morpholino-injected larvae were analyzed after labeling in the presence of recombinant human PCMT1 (rhPCMT1) and tritiated SAM by SDS-PAGE **(A,B)** followed by fluorography **(C,D)**. Two additional biological replicates are shown here for the SDS-PAGE and fluorography analyses shown in main Figure 4.

Supplementary Table 1 Primers used for qPCR, cloning of zebrafish *pcmt* and *pcmtl* coding sequences, and validation of CRISPR knockout of mouse *Pcmt1* in HT22 cells.

## Abbreviations

GCaMP6f: GFP-Calmodulin-M13 genetically encoded calcium indicator
PCMT: Protein l-isoaspartyl methyltransferase
IP_3_: Inositol (3,4,5)-trisphosphate
PTZ: Pentylenetetrazol
SAM: *S*-adenosyl methionine
PLC: phospholipase C.
