# Supplementary figures and images for: l-Isoaspartyl Methyltransferase Deficiency in Zebrafish Leads to Impaired Calcium Signaling in the Brain
Source: Front Genet. 2021 Jan 21;11:612343. doi: 10.3389/fgene.2020.612343 (PMC7859441; doi:10.3389/fgene.2020.612343)

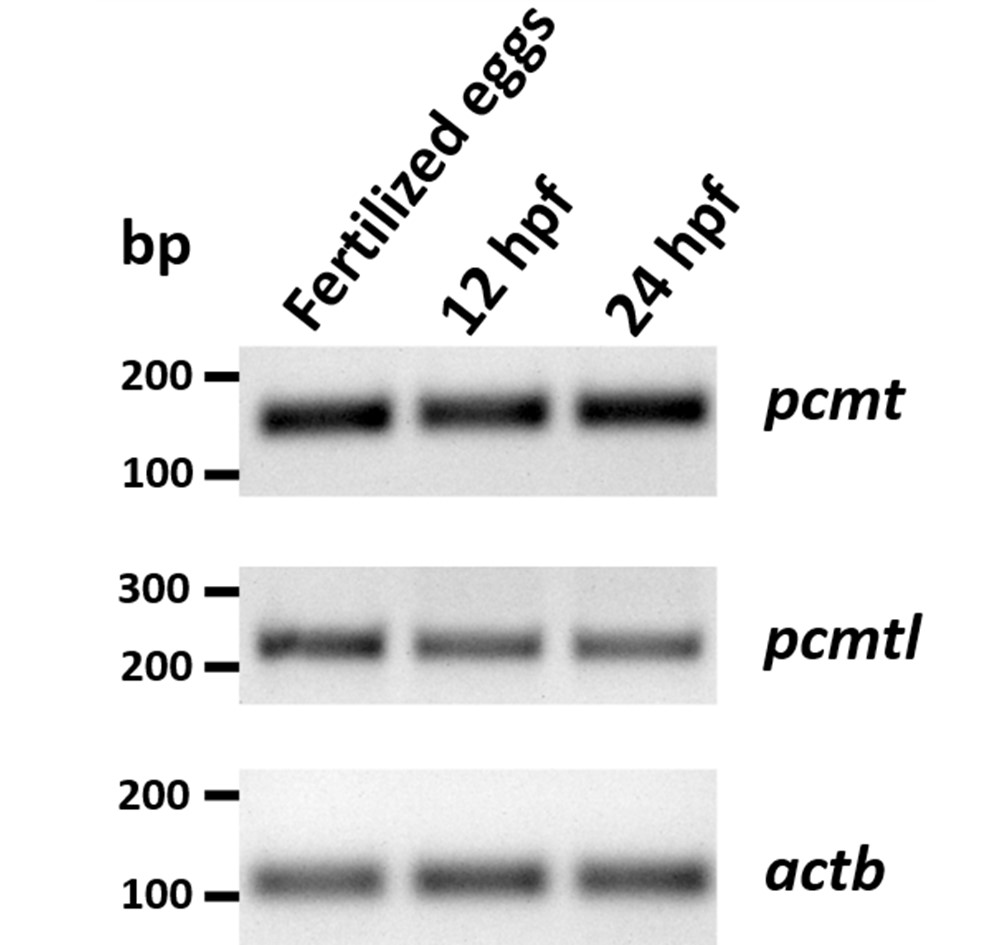

Supplement: Supplementary Figure 1 — Agarose gel showing PCR amplicons of pcmt and pcmtl from zebrafish egg and embryo cDNA. Total RNA was extracted from eggs (30 min post-fertilization) and embryos at 12 and 24 hpf for cDNA synthesis. PCR amplification using primers specific for the pcmt and pcmtl genes confirmed the presence of both transcripts at the 3 tested stages of development. Beta-actin (actb) was used as a reference gene. [file Image_1.JPEG]

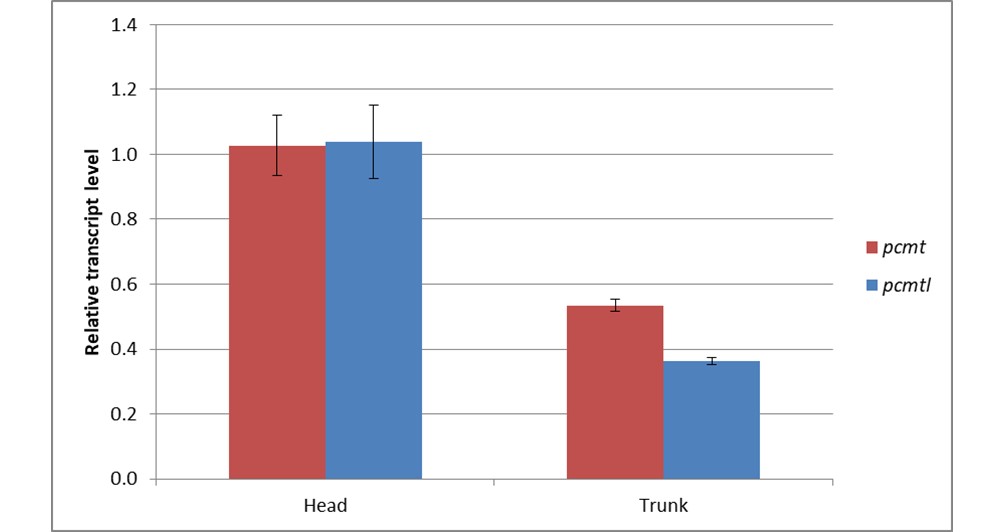

Supplement: Supplementary Figure 2 — Expression of pcmt and pcmtl transcripts in the head and trunk regions of zebrafish larvae. The head was separated from the trunk of 7 dpf zebrafish larvae using a scalpel and samples were placed directly on dry ice for RNA extraction. Relative transcript levels of pcmt (red bars) and pcmtl (blue bars) were determined by qPCR, using eef1a1l1 as reference gene. Data shown are means ± SEMs for the 3 biological replicates. Each replicate consisted of a pool of 10 head or trunk samples. [file Image_2.JPEG]

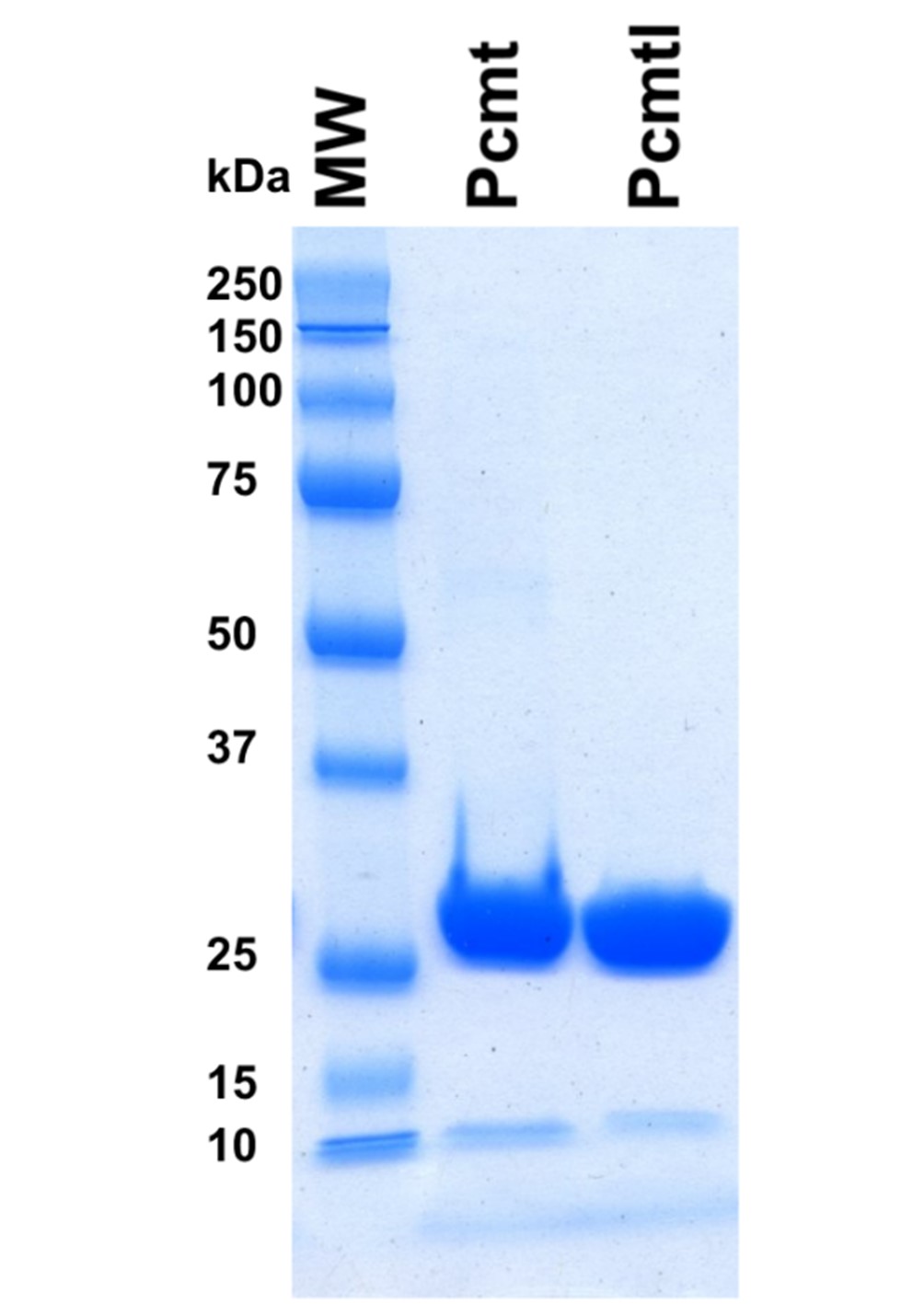

Supplement: Supplementary Figure 3 — SDS-PAGE analysis to assess the purity of recombinant zebrafish Pcmt and Pcmtl preparations. Zebrafish Pcmt and Pcmtl were expressed as N-terminal His-tag fusion proteins in E. coli (expected MW of 27 kDa for both recombinant proteins) and purified by nickel affinity chromatography followed by dialysis for desalting. For each preparation, the equivalent of 10 μg protein was loaded onto a 12% polyacrylamide gel under denaturing conditions, followed by Coomassie blue staining. [file Image_3.JPEG]

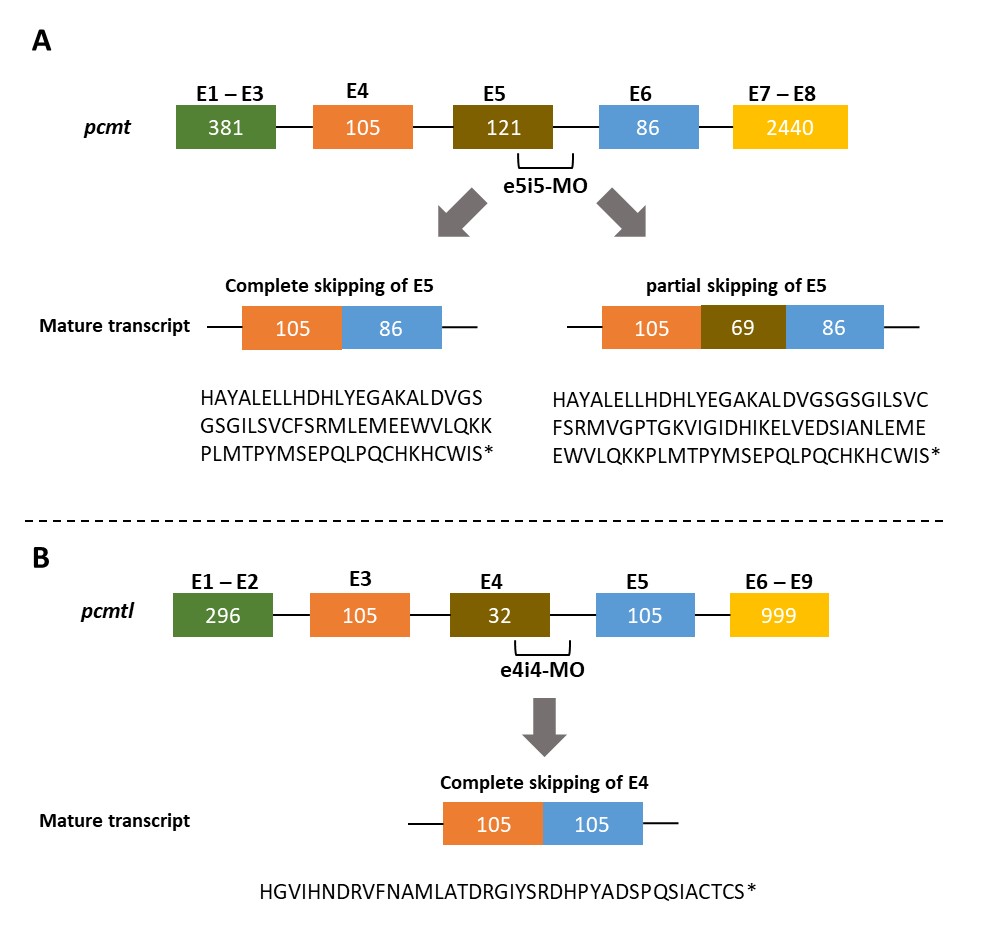

Supplement: Supplementary Figure 4 — Morpholino strategy used for knocking down pcmt and pcmtl in zebrafish larvae. (A) The pcmt e5i5 MO leads to two events, whole and partial skipping of exon 5. Complete skipping of exon 5 leads to a frameshift after amino acid 99 and an early stop codon after 132 amino acids. Partial skipping of exon 5 leads to a frameshift after amino acid 122 and an early stop codon after 155 amino acids. In both cases, the predicted truncated proteins miss the AdoMet-II, AdoMet-III and post-III domains that are required for enzyme activity. (B) The pcmtl e4i4 MO leads to skipping of the whole exon 4 leading to a frameshift after amino acid 53 and an early stop codon after amino acid 58. The resulting truncated Pcmtl protein is missing all the conserved domains that are important for the enzyme activity. Exons and corresponding numbers of nucleotides are represented by the colored boxes. Ensembl transcripts pcmt-201 and pcmtl-201 were taken as reference. [file Image_4.JPEG]

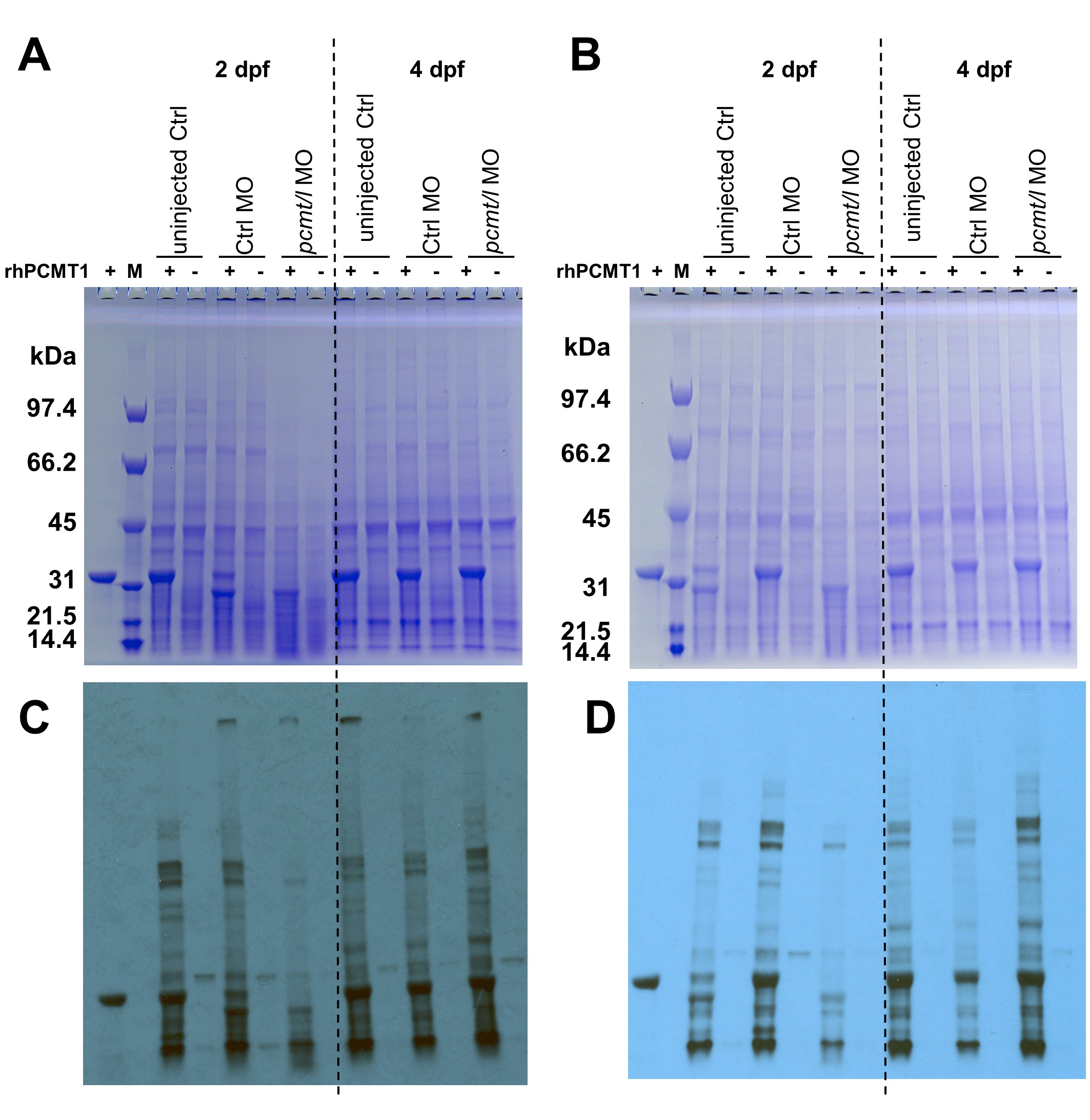

Supplement: Supplementary Figure 5 — Knockdown of pcmt and pcmtl leads to higher isoaspartyl levels in zebrafish larvae. Protein extracts of uninjected, control MO-injected and pcmt/l morpholino-injected larvae were analyzed after labeling in the presence of recombinant human PCMT1 (rhPCMT1) and tritiated SAM by SDS-PAGE (A,B) followed by fluorography (C,D). Two additional biological replicates are shown here for the SDS-PAGE and fluorography analyses shown in main Figure 4. [file Image_5.JPEG]
