# Supplementary material for: l-Isoaspartyl Methyltransferase Deficiency in Zebrafish Leads to Impaired Calcium Signaling in the Brain
Source: Front Genet. 2021 Jan 21;11:612343. doi: 10.3389/fgene.2020.612343 (PMC7859441; doi:10.3389/fgene.2020.612343)
Supplement: Supplementary Table 1 — Primers used for qPCR, cloning of zebrafish pcmt and pcmtl coding sequences, and validation of CRISPR knockout of mouse Pcmt1 in HT22 cells. [file Table_1.DOCX]

Table **S1**: Primers used for qPCR, cloning of zebrafish *pcmt* and *pcmtl* coding sequences, and validation of CRISPR knockout of mouse *Pcmt1* in HT22 cells

| Target gene | Sequence 5’ 🡪 3’ | Purpose |
| --- | --- | --- |
| *D. rerio pcmt* | FWD: CACCATGGCCTGGAAATCCGGAG  REV: TCAAAGTTCATCCCTTGACCACTG | TOPO® Cloning of *pcmt* into pET100 for recombinant protein expression. |
| *D. rerio pcmtl* | FWD: CACCATGGCATGGATGTCTAGTGGC  REV: TCAGAGCTCATCGCCCGGCCAC | TOPO® Cloning of *pcmtl* into pET100 for recombinant protein expression. |
| *D. rerio pcmt* | FWD: CCACAGACCGTTCCCATTTC  REV: GCTCCTTCATAGAGGTGGTCA | qPCR for quantification of *pcmt* transcript levels in zebrafish |
| *D. rerio pcmtl* | FWD: CATGGAGTCATTCACAACGACA  REV: TATCCACTTCCAGACCCCACA | qPCR for quantification of *pcmtl* transcript levels in zebrafish |
| *D. rerio eef1a1l1* | FWD: CTGGAGGCCAGCTCAAACAT  REV: ATCAAGAAGAGTAGTACCGCTAGCATTAC | Reference gene for qPCR analysis |
| *M. musculus Pcmt1* | FWD: AAGGATGACCCAATGCTCCT  REV: GGGCTGCTGTTTCTACTTGG | Validation of *Pcmt1* CRISPR knockout in HT22 cells |
